# *Bauhinia monandra* derived mesoporous activated carbon for the efficient adsorptive removal of phenol from wastewater

**DOI:** 10.1038/s41598-025-17444-w

**Published:** 2025-08-29

**Authors:** Bhojaraja Mohan, Chikmagalur Raju Girish, Gautham Jeppu, Praveengouda Patil

**Affiliations:** Department of Chemical Engineering, Manipal Institute of Technology (MIT), Manipal Academy of Higher Education (MAHE), Manipal, Karnataka 576104 India

**Keywords:** *Bauhinia monandra*, Porous carbon, Isotherm, Isosteric heat, Response surface methodology, Environmental sciences, Engineering

## Abstract

The quality evaluation of the activated carbon made from agricultural waste is investigated in the adsorption of phenol. Adsorbent was prepared through the activation of orthophosphoric acid, producing a structure that is porous with a surface area of 1090 m^2^/g. Elemental composition analysis confirmed that the carbon composition is 78.23%. X-ray photoelectron spectroscopy (XPS) and other characterizations were conducted to study the structural, chemical and thermal performance metrics. The results from the Boehm titration were aligned with the point of zero charge. Response Surface Methodology was used to optimize adsorption parameters. The adsorption outcomes demonstrated the effectiveness of phenolic removal, with the Langmuir isotherm under optimal conditions exhibiting a maximum adsorption capacity of 185.93 mg/g. The study on the kinetics of adsorption showed that they closely matched the second-order pseudo model (R^2^ > 0.98). Thermodynamic studies indicate that adsorption occurs spontaneously, as evidenced by negative ΔG° values. This further validates its exothermic nature and decreases randomness at the solid–liquid interface, as indicated by negative ΔH° and ΔS° values. The adsorbent regeneration and spiking studies were methodically examined. Activation energy and isosteric heat of adsorption were assessed. The adsorption mechanism is discussed thoroughly. These results highlight the effectiveness of activated carbon as a practical adsorbent for treating wastewater.

**Supplementary Information:**

The online version contains supplementary material available at 10.1038/s41598-025-17444-w.

## Introduction

Environmental pollution is a major global issue, encompassing the contamination of air, water and land. The fast pace of development of the city, industrial growth, and rising population has greatly improved the number of pollutants released into the environment. This surge in pollutants represents a substantial risk to wildlife, public health condition, and natural ecosystems^1^. Water contamination by chemicals has grown to be a significant problem and concern for government organisations, industries, and society at large^2^. Industrial effluents frequently comprise an extensive range of toxic contaminants, such as organic pollutants, heavy metals, drugs, pesticides, and various dyes including phenols^3^. Phenol is an organic molecule having the chemical formula C₆H₅OH, contains a hydroxyl (-OH) group attached to a benzene ring. The melting point of phenol is 40.5 °C, while its boiling point is 181.7 °C^4^. The pharma companies, petrochemicals, oil refining, cookery, resin production, plastics, paper, paint, pulp, and wood goods are just a few of the numerous industries that release effluents that contain phenolic composites. If these phenolic compounds released untreated, it can pose risks to human health, animal life, and aquatic environments. Globally, more than six million tonnes of phenol are generated each year, and this amount is continuously rising^5^. The reported levels of phenol concentration in various industrial wastewater are shown in Table 1.

**Table 1:** The reported levels of phenol concentration in various industrial wastewater^13^.

Industry	Phenol concentration (mg/L)
Paint and rubber manufacturing	1.1–10
Ferrous	5.6–9.1
Pulp & Paper	22
Textile (Dyeing/Finishing)	100–150
Petroleum Refining & Petrochemical	40–1220
Phenolic resin production	1200–1600
Coal conversion	1700–7000

Phenol is a highly toxic, mutagenic and carcinogenic pollutant with widespread adverse effects on human health, aquatic ecosystems, terrestrial animals, plants, and groundwater^6^. In the process of treating wastewater, phenol is regarded as a priority contaminant. The World Health Organization (WHO) and the United States Environmental Protection Agency (U.S. EPA) have set the maximum allowable limits for phenol in wastewater at 1 mg/L and in consuming water at 0.1 mg/L^7^. Furthermore, National Pollutant Release Inventory (NPRI) and the U.S.EPA rank phenol as the 11^th^ of 126 harmful substances^8^. Acute exposure of phenol pollutant in humans through ingesting, inhalation, or skin contact can cause severe burns, necrosis, irritation of the mucosa, and systemic toxicity, which includes cardiovascular collapse, muscular spasms, and depression of the central nervous system (CNS), effect vital organs like the kidney, liver, and immunological system. Long-term exposure has been associated with neurological symptoms, reproductive damage, hepatic and renal failure, and perhaps endocrine disruption. Additionally, the individuals had dark urine, vertigo or dizziness, unexplained weight loss, and even a discernible decrease in appetite^9^. Phenol can easily enter groundwater and surface waters like rivers and lakes due to its high solubility in water and short half-life in soils. A wide range of livestock, including fish, amphibians, algae, and invertebrates, are at risk of acute poisoning from phenol in these circumstances, which can have negative effects on photosynthesis, growth, and reproduction^10^. By preventing the growth and activity of microbial populations necessary for normal biodegradation processes, phenol can reduce dissolved oxygen levels and change the pH of water, even at low concentrations, destabilising aquatic ecosystems^11^. Additionally, it may exist in aquifers and move with groundwater flow, contaminating drinking water and endangering the health of people and the environment. Particularly vulnerable is shallow groundwater close to pollution sites^12^. Plants may absorb phenol from the soil, which could result in reduced growth, oxidative stress-induced cellular damage, and impaired seed germination. Animals on land that are exposed to phenol-contaminated habitats may experience developmental delays, neurological disorders, and reproductive troubles. Due to increased concern about phenolic contaminant, researchers are looking for the effective and the sustainable ways to remove phenol from wastewater. This has been a major area of focus in the water treatment industry, intending to create efficient and environmentally beneficial methods.

To address the adverse effects of pollutants in the waste water, various treatment approaches, including chemical, physical, and biological methods, have been investigated to eliminate solid particles^14^. Among these, physical methods such as sedimentation and filtration are considered cost-effective. However, these methods are not efficient for removing dissolved pollutants and require additional treatment processes. In contrast, chemical processes like oxidation, flocculation, and coagulation are more effective at removing pollutants, but they tend to be expensive, energy-intensive, and produce sludge^15^. Implementing biological treatments on a large scale is challenging due to slow degradation rates, difficulties with a variety of pollutant compounds, and are highly sensitive to environmental conditions, even though they are environmentally friendly. On the other hand, adsorption is recognized as effective method for removing impurities from wastewater. This method is commended for its versatility, ease of use, affordability, potential for reusing adsorbents, and environmental friendliness. As a result, adsorption has been extensively researched and is frequently applied in practical situations^16^.

The choice of adsorbents in adsorption depends on the particular application and the kinds of contaminants involved. Activated carbon is a commonly employs adsorbent, which is favoured for its significant surface area and porous character. This makes it extremely effective at adsorbing a broad variety of pollutants^17^. Although zeolites, silica gel, activated alumina, clay minerals, and other adsorbents have advantageous adsorption qualities^18^. Different types of adsorbents are used to treat wastewater apart from activated carbon, and each has its own special qualities, benefits, and drawbacks. Clay minerals, such bentonite and kaolin, are cheap and widely available^19^. They are good at eliminating some heavy metals and dyes, but they are not very good at removing organic contaminants and can cause sludge generation^20^. Although industrial by-products like fly ash and red mud offer easily accessible, affordable alternatives that can adsorb specific colours and heavy metals, their limited use is due to their variable quality and potential for secondary pollution through leaching^21^. Zeolites are crystalline aluminosilicates with porous structures that have good ion-exchange properties and great selectivity for metal and ammonium ions, but they are less efficient for complex organic compounds that have not been altered^22^. A very porous type of aluminium oxide, activated alumina is useful for eliminating pollutants including fluoride, arsenic, and nitrate, but its ability to remove organic pollutants is constrained^23^. Silica gel has a limited capacity for nonpolar organics and is mainly employed for the adsorption of moisture and certain metal ions. It may need modification or combination with other materials for enhanced efficiency^24^. Among these, activated carbon, particularly when derived from agricultural waste is distinctive due to its large surface area, remarkable adsorption capacity for a wide range of contaminants, including dyes, heavy metals, and emerging pollutants, quick adsorption kinetics, and capacity for regeneration and reuse^25^.

Activated carbon is frequently used in wastewater treatment to efficiently remove a variety of organic and inorganic contaminants. Its porous structure allows for quick adsorption kinetics, which speeds up and improves the efficiency of the process. Activated carbon is stable both chemically and thermally at different pH and temperature levels. By utilising renewable biomass resources, agricultural waste-based activated carbon not only performs on par with or better than commercial activated carbon, but it also offers increased cost-effectiveness and environmental sustainability. Because of its wide range of applications, steady high efficiency, and low chance of secondary contamination, it is the most dependable and ideal adsorbent for thorough wastewater treatment^26^. Activated carbon’s eco-friendliness and capacity to be made from renewable resources, like agricultural waste, cost effectiveness, highlight its significance as an adsorbent in tackling environmental issues. These characteristics make activated carbon a vital component for various commercial, industrial, and environmental applications, including air filtration and water treatment^27^. Agricultural wastes such as sawdust, rice husks, coconut shells, tree pods, etc. are converted into activated carbon, which boosts rural economies and lessens reliance on non-renewable resources. Because of its regenerative and reusable nature, the technique is economical, reduces solid waste, and encourages sustainable, local production^28^. Activated carbon effectively removes harmful contaminants such as heavy metals, phenols, and pharmaceutical residues from water, which helps to reduce the risk of waterborne diseases and long-term health problems which directly contributes to SDG 3: Good health and well-being. By eliminating contaminants from wastewater, activated carbon supports SDG 6: Clean water and sanitation by rendering it safe for discharge and reusable. Activated carbon are effectively included into treatment systems such as fixed-bed columns and batch reactors. Its seamless integration with low-cost, AI-optimized, decentralised technologies enables scalable, modified variations permit selective adsorption and sustainable solutions, expanding SDG 9: Industry, Innovation, and Infrastructure. Activated carbon safeguards aquatic life and biodiversity by avoiding the release of harmful pollutants into water bodies. In support of SDG 14: Life below water which contributes to ecological balance.

Using agricultural waste to produce activated carbon is essential since it has both financial and environmental advantages. By transforming possible waste into useful adsorbents, using the abundant agricultural waste as a raw substance for to prepare activated carbon solves waste management concerns and offers a more affordable option to conventional materials. One promising method for producing activated carbon that has enhanced adsorption capabilities for phenolic compounds is to investigate different biomass sources with unique properties, such as the pods of the *Bauhinia monandra* tree. These trees are found in tropical and subtropical regions worldwide, including Southeast Asia, Indian subcontinent, and South America. Some studies have already examined the usage of pods from different trees for producing activated carbon^29^.

To the best of authors’ knowing, no research has been carried out on the usage of *Bauhinia monandra* tree pods to remove phenol. The production, characterisation, and usage of adsorbent made from these pods for phenol elimination are the main objectives of this study. The optimisation parameters were established using the response surface methodology. To comprehend the adsorption mechanisms, thermodynamic, kinetic, and isotherm investigations were conducted. Additionally, this research includes a comprehensive evaluation of desorption and regeneration factors, as well as studies involving spiked samples.

## Materials and methods

### Materials

*Bauhinia monandra* tree pods were collected from the MAHE campus and surrounding areas in Manipal, Karnataka, India. Phenol (C_6_H_5_OH), sodium hydroxide (NaOH), sodium bicarbonate (NaHCO_3_), orthophosphoric acid (H_3_PO_4_), hydrochloric acid (HCl), sodium carbonate (Na_2_CO_3_) and sodium chloride (NaCl), analytical reagent quality chemicals acquired from Merck India limited.

### Equipment

Various equipment used for the synthesis of the adsorbent and for carrying out adsorption experiments are Hot air oven (KEMI, India), muffle furnace (REMI- India), orbital shaking incubators (Remi CIS-24 Plus, India), UV–Vis Spectrophotometer (Shimadzu-UV 1900i-Japan).

### Adsorbate solution preparation

The appropriate amount of phenol was dissolved in one litre of distilled water to prepare a stock concentration of 1000 mg/L. To achieve a homogeneous solution, the mixture was continuously stirred until all the phenol crystals were completely dissolved. This stock was then diluted for further investigation. Phenol adsorption is examined using the UV–Visible Spectrophotometer, wavelength (λ_max_) of 270 nm. The device has a detection limit (LOD) of 0.14 mg/L and a quantification limit (LOQ) of 0.41 mg/L. The equipment is calibrated before use. The absorbance vs wavelength plot of degradation of phenol is shown in supplementary file Fig. S1.

### Synthesis of activated carbon

A systematic procedure was utilized to synthesise activated carbon from *Bauhinia monandra* tree pods. Initially, the pods were collected from the Manipal locality in Karnataka and cleansed completely with distilled water in order to eliminate the dust and leftover impurities. After cleaning, the pods were dried for 24 h at 105 °C in an oven. The outer shell was then removed from the cleaned pods, which were pulverized into a fine particle and sieved to eliminate any particles smaller than 425 μm. The sieved powder was dried for an additional 60 min to ensure complete moisture removal. For chemical activation, H_3_PO_4_ was added to the dried sample at a 1:1 (vol/mass) ratio, allowing for consistent activation. To create a porous structure, the impregnated sample was subsequently carbonised for 120 min at 600 °C in an inert atmosphere^26^. Following carbonization, the activated sample remained allowed to cool to the necessary temperature before being cleaned and its pH brought closer to neutral using a 1% sodium bicarbonate (NaHCO_3_) solution^30^. After being cleaned, the activated carbon was subsequently dried at 105 °C for 12 h and marked as *Bauhinia monandra* tree pods activated carbon (BTPAC) to preserve its quality while being kept in an airtight container.

### Yield percentage of activated carbon

The Percentage yield measures carbon efficiency, indicating how much material remains after activation and carbonization. The yield percentage is shown in Eq. (1)^31^.

1 $$\%Y = \frac{{W}_{f}}{{W}_{i}} \times 100$$

The $\%Y$ is the yield percentage, ${W}_{i}$ is the initial weight (before processing), ${W}_{f}$ is the final weight (after processing).

### Adsorbent (BTPAC) characterisation

Characterizing activated carbon is essential for understanding how its structural, morphological, and surface chemical properties influence its adsorption performance. The prepared activated carbon’s surface area, crystallinity, functional groups, pore size distribution, and stability in terms of both chemical and thermal factors were assessed in order to properly evaluate its properties.

The Brunauer–Emmett–Teller (BET) surface area, hysteresis and isotherm types were identified using N_2_ adsorption–desorption data at 77 K using a Quantachrome® ASiQwinTM surface area analyser. The outgassing time was approximately 2 hours^32^. Furthermore, the Barrett-Joyner-Halenda (BJH) method was used to derive the pore volume and pore size distributions, which offer detailed insights into the material’s porous structure^33^. Proximate analysis was conducted as per the American Society for Testing and Materials (ASTM)^34^. The ultimate analysis provided insight into the material’s elemental composition (C, H, O, N, and S) by ELEMENTAR Vario EL III. X-ray diffraction (XRD) analysis was carried out using an X-ray diffractometer—Rigaku minifex 600 5th gen, 2θ values between 5° and 80° to analyse the amorphous features of the BTPAC^33^. The adsorbent’s morphology was examined for both before and after adsorption using a high-resolution—field emission scanning electron microscope (HR-FESEM) by GEMINI SEM 300, Carl Zeiss, Germany. The Energy Dispersive X-ray Spectroscopy (EDS) analysis is carried out to determine the elemental composition of BTPAC. To perform thermogravimetric analysis (TGA), a TA 55-discovery from TA Instruments (Austria) was used. Analyses were carried out at a rate of heating of 10 °C/min starting at room temperature to 800 °C^35^.

The Boehm titration technique was used to identify the basic and acidic functional groups present on the surface of BTPAC. The technique used was titration with HCl to assess basic groups and titration with various bases (NaOH, Na₂CO_3_, and NaHCO_3_) to measure the concentration of acidic functional groups^36^. The batch equilibrium (drift method) approach was used to find the activated carbon’s point of zero charge (pHpzc). To learn more about how the adsorbent interacts with charged species in aqueous solutions^37^. Fourier Transform Infrared spectroscopy (FTIR) was used to identify the functional groups on the surface of the adsorbents. The spectra were recorded with an ATR-FTIR Shimadzu-8400 s. The spectrum was obtained over the array of 400 to 4000 cm^−1^^38^. The X-ray Photoelectron Spectroscopy (XPS) results of BTPAC’s pre and post phenol adsorption were assessed to recognize the bonding types and surface characteristics^39^. It is analysed by Thermofisher Nexsa XPS with an Al Kα radiation source. The measurements were conducted under an ultra-high vacuum of 10^−8^ mbar, 200 eV of pass energy and 10 ms of dwell time.

### Optimisation of operating parameters for phenol adsorption

Important parameters were selected to optimize the adsorption process depending on preliminary studies and a review of the literature. These parameters include equilibrium time, BTPAC dosage, solution pH, solution temperature, and initial concentration. A standard conventional technique was used to examine equilibrium time (min). The remaining optimization parameters for the adsorbent were improved using response surface methodology, which involved Design-Expert application to evaluate key factors such as dosage of the BTPAC (g/L), initial concentration (mg/L), pH, and temperature (°C). The percentage of phenol removed was used to characterize the response^40^. Response surface methodology (RSM) is an advanced technique that integrates statistical and mathematical methods with experimental design to optimize the removal of phenol or other pollutants^41^. RSM efficiently manages several factors by creating a quadratic model, which makes it possible to forecast the ideal circumstances for the highest removal efficiency. RSM’s practical utility is exemplified by the optimization process conducted using Design-Expert software, which offers an intuitive interface for model validation and modification.

### Adsorption experiment

A standard batch study was investigated to ascertain the equilibrium time for the percentage removal of the adsorbate. In this procedure, a fixed dosage of BTPAC was mixed into a solution with a known initial concentration of the adsorbate, while continuously stirring and maintaining all other adsorption parameters constant. Samples were collected at predetermined time intervals in clean, dry vials after the trials were completed. Additionally, materials were centrifuged to minimize particle interference prior to analysis. A graph was created to plot the removal percentage against contact time, with the equilibrium time identified as the point at which there will be no significant enhancement in adsorption. This equilibrium time was then utilized in all subsequent optimization experiments. With the use of Design-Expert applications, RSM was employed to optimise the process parameters, including dose, pH, temperature, and initial concentration, once the equilibrium time was established. Table 2 exhibits the ranges and levels of the independent parameters utilized in the RSM.

**Table 2:** The ranges and levels of the independent parameters utilized in the RSM.

Parameters	Code	Levels of independent parameters		
		− 1	0	1
Dosage (g/L)	A	0.6	1.18	1.8
pH	B	4	6.5	9
Temperature (°C)	C	25	35	45
Initial concentration (mg/L)	D	20	30	40

In the current study, four adsorption variables were examined, leading to a design matrix with 30 experiments. This had 6 duplicates, 8 axial, and 16 factorial points. Replication was utilized to determine the experimental errors. A 50 mL adsorbate solution with a predetermined initial concentration was made in a 250 mL conical flask for each run produced as per Design-Expert program, and the appropriate dosage was added. The pH of the solution had been adjusted to the required amount using either 0.1 N HCl or 0.1 N NaOH. Subsequently, the flasks were kept at the appropriate temperature for the predefined equilibrium time while being supported on an orbital shaker. Once the shaking is completed, the solutions were filtered, and the residual concentration of phenol was measured. The removal efficiency and adsorption capacity $\left({q}_{e}\right)$ were calculated using Eqs. (2) and (3), respectively^27,42^.

2 $$Removal\, (\%)=\frac{(Co-Ce)}{Co} * 100$$

3 $$\text{Adsorption capacity }({\text{q}}_{\text{e}})=\frac{(\text{Co}-\text{Ce})}{\text{m}} *\text{ v}$$

In which, $Co$ phenol solution’s initial concentration of (mg/L), $Ce$ the final concentration (mg/L) of phenol pollutant at equilibrium, *m* is mass of BTPAC (g), and volume (L) of pollutant is represented as $v$.

#### Isotherm, kinetics and thermodynamic studies

Isotherm investigations were carried out with optimal BTPAC’s dosage, pH, temperature, and contact time. During the trials, initial pollutant concentrations were increased from 10 to 1000 mg/L, with all parameters remaining constant until equilibrium was achieved. The Langmuir, Redlich–Peterson, Temkin, Dubinin-Radushkevich, and Freundlich isotherm models were applied to examine the surface characteristics and adsorption mechanism. Analysis of non-linear regression was utilized to calculate model parameters, assess the error, and compute the correlation coefficient for the best-fitting model. The separation factor $${(R}_{L}$$), was used to assess the adsorption process’s favourability using the Langmuir constant $${(K}_{L})$$ and initial concentration ($$Co)$$. The supplementary file table S1 contains a list of adsorption isotherm models.

Kinetic adsorption studies were carried out using BTPAC to investigate the rate of adsorption and the mechanism of phenol pollutant on adsorbent. The initial concentrations varied from 30 to 150 mg/L in order to assess the removal behaviour, while the other parameters remained unchanged. Time-dependent adsorption studies were carried out under optimal conditions by taking samples at predetermined intervals until equilibrium was achieved. The data from these trials were modelled using non-linear forms of kinetic equations. The list of equations models for adsorption kinetics is presented in the supplementary file table S2.

#### Evaluation of activation energy from adsorption kinetics rate constant

Kinetic adsorption experiments were conducted at varied temperatures between 25 and 45 °C in order to determine the activation energy ${(E}_{a}$). The Arrhenius equation was employed to evaluate activation energy, representing the minimum energy required for the reaction to proceed^52^. ${E}_{a}$ was determined from the slope between $ln{ K}_{2}$ and $1/T$. Arrhenius equation (linearised) is shown in the Eq. (4)^53^.

4 $$\mathit{ln}{\mathit{ K}}_{2}=\mathit{ ln}A-\frac{{E}_{a}}{RT}$$

wherein ${K}_{2}$ is the rate constant from pseudo-second order (PSO) in (g/mg. min), and A is the Arrhenius factor, $R$ is the universal gas constant and $T$ is temperature ($K$).

### Evaluation of model fitting in adsorption studies

Evaluating the goodness of fit for kinetic and isotherm models is crucial in adsorption studies to ensure accurate interpretation of experimental data. The sum of squared errors (SSE), chi-square (${\chi }^{2}$), and coefficient of determination (${R}^{2}$) are the three metrics most frequently employed statistical tools to evaluate model fitting.

The ${R}^{2}$ value indicates how effectively the model describes the variability in the experimental values. It varies from 0 to 1, where a value near to 1 suggests an excellent fit, meaning that the model successfully describes the adsorption behaviour^54^. It is calculated using the Eq. (5).

The ${\chi }^{2}$ value and SSE assess the difference between experimental and predicted values^55^. A lower ${\chi }^{2}$ value suggests a better fit because it signifies minimal deviation between observed and modelled adsorption capacities^54^. It is computed as Eqs. (6) and (7).

5 $$R^{2} = 1 - \frac{{\sum \left( {q_{e.\exp } - q_{e.\bmod el} } \right)^{2} }}{{\sum \left( {q_{e.\exp } - \overline{q}_{e.\exp } } \right)^{2} }}$$

6 $${\upchi }^{2}= \sum_{i=1}^{n}\frac{{({q}_{e.exp}-{ q}_{e.model})}^{2}}{{ q}_{e.model}}$$

7 $$\text{SSE}=\sum_{i=1}^{n}{({q}_{e.exp}-{ q}_{e.model})}^{2}$$

where the experimental adsorption capacity is represented by ${q}_{e.exp}$, The predicted value from the model is ${q}_{e.model}$, and $\overline{q}_{e.\exp }$ is the average of experimental values.

The thermodynamic study investigated the effect of adsorption temperature (15, 25, 35, and 45 °C) on the phenol adsorption. To better understand the thermodynamic adsorption process, it is crucial to evaluate key thermodynamic characteristics related to the movement of a single mole of the solution’s solute phase to the interface between the solid and liquid phases^56^. The parameters include standard entropy denoted as *ΔS*°, standard enthalpy denoted as *ΔH*°, and standard Gibbs free energy denoted as *ΔG*°^7^. Furthermore, the isosteric heat of adsorption $(\Delta Hx$*)* is determined by the equation of Clausius-Clapeyron by plotting $ln{C}_{e}$ vs $1/T$ on a constant or fixed adsorption loading^57^. The standard equations of (8)–(11) thermodynamic parameters are mentioned below.

8 $$\Delta {G}^{o} = - RTln{K}_{d}$$

9 $${K}_{d}=\frac{{C}_{a}}{{C}_{e}}$$

10 $$\Delta {G}^{o} = \Delta {H}^{o}-T\Delta {S}^{o}$$

11 $$\frac{d ln{C}_{e}}{dT}= \frac{-\Delta Hx}{R{T}^{2}}$$

In the above equations, ${C}_{a}$ represents equilibrium adsorption amount (mg/L), ${C}_{e}$ is the phenol concentration on the adsorbent at equilibrium (mg/L) and ${K}_{d}$—distribution coefficient.

#### Desorption-regeneration analysis of the adsorbent

Desorption experimentations were conducted to assess the reusability of the BTPAC. This procedure involved adding 2 g of the activated carbon at a phenol concentration of 50 mg/L to 100 mL of solution. The mixture was continuously mixed for 3 h at 25 °C. Once the adsorption process is complete, the adsorbent is carefully removed from the solution using decantation to recover the phenol-loaded adsorbent. The recovered adsorbent was dried, subsequently exposed to a 1 mol/L NaOH solution to aid in the adsorbate’s desorption from the BTPAC surface^58^. NaOH solution helps to break the adsorptive interactions among phenol and the activated carbon, allowing the pollutant to be released back into the solution. The entire adsorption–desorption process was repeated to determine the reusability efficiency. After treatment, the adsorbent was dried for 60 min in an oven at 105 °C to eliminate any remaining moisture and prepare it for the subsequent adsorption cycle.

#### Spiked studies

Phenolic contaminants were added to real water samples to evaluate the effectiveness of BTPAC as an adsorbent. The goal of this procedure was to assess the adsorbent’s performance in removing impurities from water sources. Samples of distilled water, lake water, river water, tap water, and seawater were gathered from an array of sources situated nearby. A known concentration of phenol was introduced by spiking the real water with a prepared phenol stock solution. To prevent contamination, these samples were stored in sterile containers. Subsequently, the adsorption investigation was carried out under ideal circumstances to make sure appropriate results^59^.

## Results and discussions

### Yield percentage

The yield of adsorbent produced from the *Bauhinia monandra* tree pod was measured at 37%, based on the precursor’s initial mass. A total of 20 mg of raw material was used to create BTPAC, resulting in the production of 7.4 mg of BTPAC after the carbonization. The use of H_3_PO_4_ as the activating agent explicates the high yield of the adsorbent. This agent improved both the penetration and retention during the process, leading to enhanced pore formation and expansion^31^.

### Characterization analysis of adsorbent

**Fig. 1:**
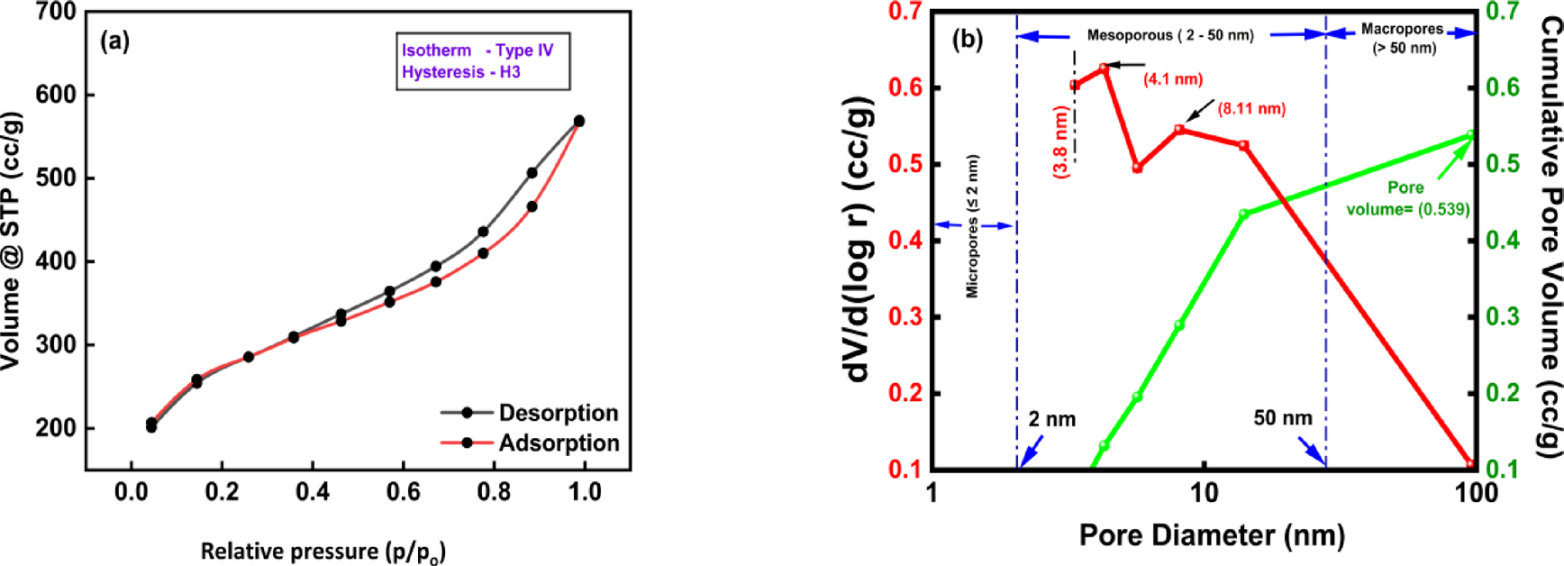
(**a**) N₂ adsorption–desorption curve, (**b**) Pore volume and pore size distribution curves.

The prepared adsorbent has a surface area of 1090.38 m^2^/g. Based on the BET isotherm N_2_ -adsorption–desorption technique was utilized to examine the porous architectures of the produced adsorbents. Furthermore, the BJH method was employed to evaluate the size distribution of pores as shown in Fig. 1.

According to IUPAC’s classification of hysteresis loops, the nitrogen adsorption–desorption graph in Fig. 1a corresponds to isotherm type IV and an H3 type hysteresis^60^. The type of isotherm reveals the several mesoporous constituents of pores within 2–50 nm^61^. The pores of the adsorbent are categorized into three types based on their diameter: micropores (< 2 nm), mesopores (in between 2 and 50 nm), and macropores (> 50 nm)^62^. The H3 type hysteresis loop shows the narrow displayed curve where a gradual increase in the slope in the adsorption–desorption curve at a high relative pressure indicates rapid adsorption without distinct saturation, suggesting the presence of wedged shape pores open at both ends or groove-like pores^63^.

The distribution pore size of BTPAC using BJH technique is illustrated in Fig. 1b, indicating that it is mesoporous, as it shows two distinct peaks, between 2 and 50 nm, which is considered a bimodal particle distribution with the highest peaks situated at 4.1 nm, 8.11 nm. It suggests that the majority of the pores have a diameter of approximately 4.1 and 8.11 nm^64^. Considering that phenol pollutants having the molecular size which ranging from 0.43 to 0.57 nm^65^, and the pore size of BTPAC is larger, indicating that phenol molecules can easily access the pores. Additionally, Fig. 1b illustrates that the pore volume of adsorbent BTPAC is 0.539 cm^3^/g. This value reflects the cumulative volume of all pores per unit mass of activated carbon. The combination of trimodal pore size distribution and substantial pore volume suggests that the adsorbent not only offers higher surface area for adsorption but also facilitates efficient mass transfer through mesopores.

### Proximate analysis and ultimate analysis of BTPAC

The proximate analysis measures the quantity of moisture present in BTPAC, with volatile compounds, ash components and fixed carbon. The used material is good for preparing the adsorbent for adsorption applications because of its fixed carbon concentration of 46.30%. This analysis demonstrates that the used agricultural waste is an appropriate or suitable material for preparing activated carbon because of its high levels of volatile compounds and low residual ash. Specifically, the material has an ash content of 9.6%, a volatile compound percentage of 32.6%, and a moisture content of 11.5%. While a low inorganic content is necessary to produce a product with low ash and high fixed carbon, a high quantity of volatile compounds usually lowers the solid yield during the carbonisation process^66^.

The ultimate analysis reveals that BTPAC consists 27.55% of oxygen, 6.2% of hydrogen, 3.41% of nitrogen, and 60.09% of carbon. This composition indicates that BTPAC has a porous structure, which provides additional active locations for the uptake of pollutants. The activated carbon’s adsorption efficiency is greatly increased by its high carbon content. Additionally, the presence of functional groups containing hydrogen, nitrogen, and oxygen improves the material’s ability to interact with and retain impurities, thereby enhancing its overall performance in adsorption applications^67^.

### XRD analysis of BTPAC

**Fig. 2:**
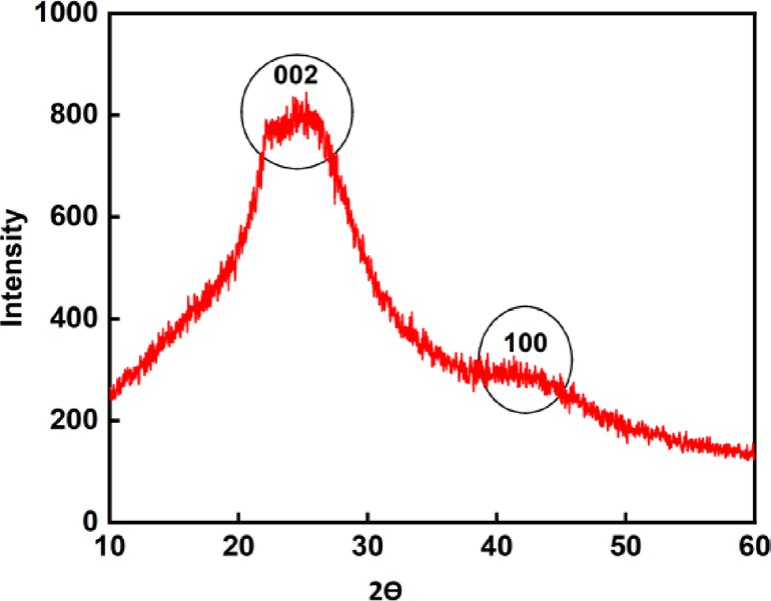
XRD patterns of BTPAC.

XRD analysis showed a broad diffraction background with peaks observed at 2θ = 25.5° and 43.5°. The absence of well-distinct peaks suggests that the structure is mainly amorphous, as illustrated in Fig. 2. These peaks correspond to the graphite planes at indices of 002 and 100^68^. Amorphous materials generally possess greater surface area per unit mass, enhancing the interaction among the adsorbent along the substrate, frequently resulting in to increased adsorption^34^. Activated carbon typically consists of three structural components: disorganized carbon, microcrystalline carbon and single-crystal-plane carbon^69^.

An amorphous texture is generally favoured for adsorption applications because it offers more open spaces, which facilitates the flow and interaction of adsorbate molecules with the material (47).

By using Scherrer’s equation, the crystallite size of BTPAC was determined^70^ as shown in Eq. (12):

12 $$S_{c} = \frac{K\lambda }{{\beta \cos \theta }}$$

where ${S}_{c}$—crystallite size, $\lambda$—wavelength of X-ray, $K$—shape factor (0.94), and $\beta$—Full breadth at half maximum in radians.

Crystalline size is approximately 1.24 nm, suggesting that BTPAC may be a in size of small crystallite, having graphitic character. Crystalline size is a small region inside the particle where the crystal lattice is placed.

### FESEM & EDS analysis of BTPAC

**Fig. 3:**
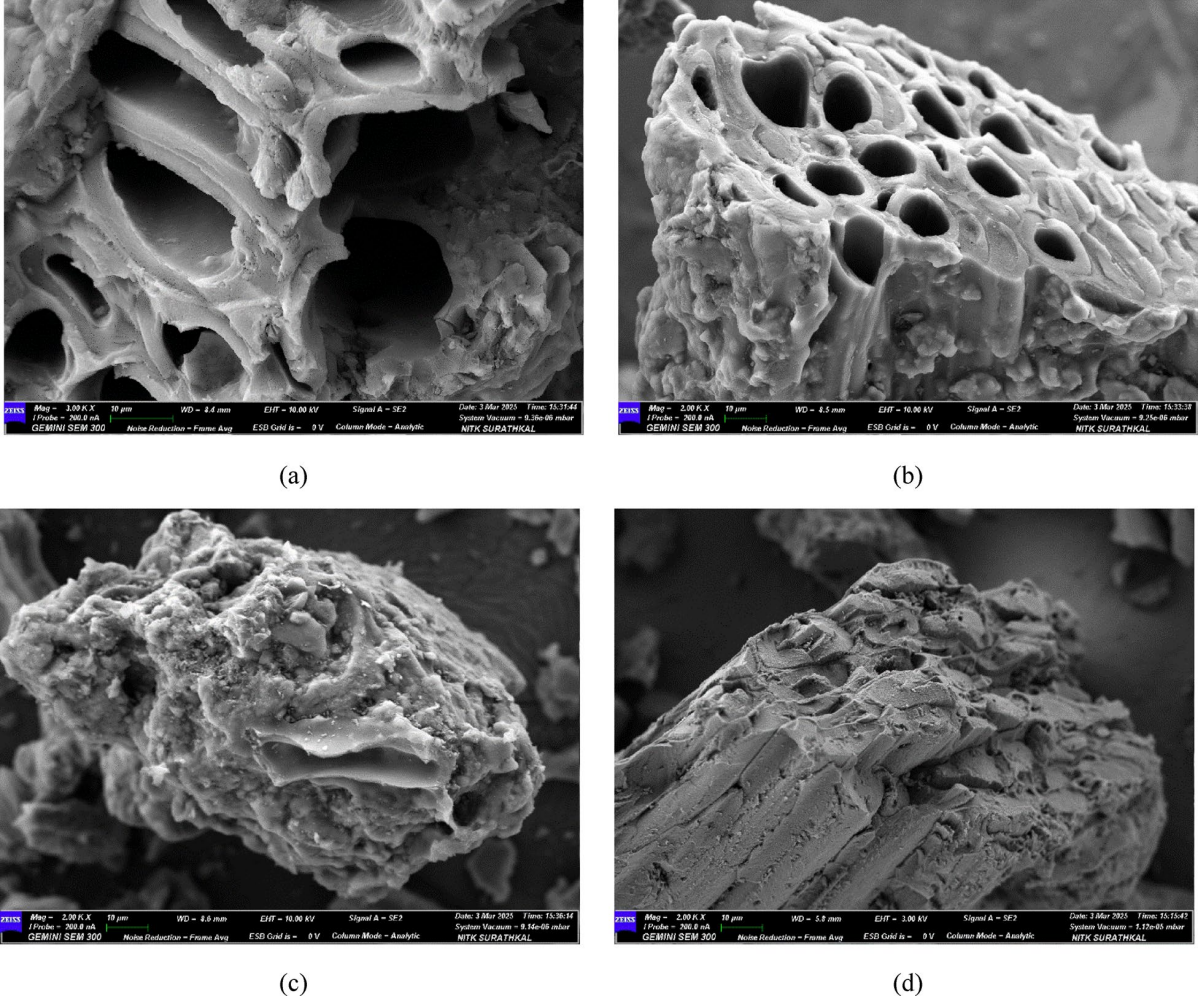
Morphological characterization of BTPAC (**a**–**d**) FESEM -before and after adsorption.

The morphological and elemental analysis of phenol adsorption on BTPAC was analyzed using high-resolution field emission SEM (HR-FESEM) as illustrated in Fig. 3. Results reveal that the BTPAC’s surface had a heterogeneous, fractured structure with big, honeycomb-like pores prior to adsorption. The surface appeared rough and featured lighter areas in the images. This suggests that significant devolatilization occurred during preparation, resulting in the release of a substantial amount of volatile matter due to the breakdown of organic components in agricultural wastes. The adsorbent precursors with phosphoric acid contain a complex mechanism characterized by several chemical reactions (48). Phosphoric acid plays a crucial role in breaking down lignocellulosic components during the impregnation and heat activation processes. This breakdown leads to the development of a porous carbon, which greatly influences the pore configuration, surface area, with adsorption efficiency. The occurrence of empty spherical pores, as shown in Fig. 3a,b prior to phenol adsorption, indicates an extremely porous surface with a significant surface area for phenol absorption. The phenol adsorption causes an extreme modification in the surface’s form, as revealed in Fig. 3c,d. The presence of flaky patches indicates that the phenol molecules have successfully bonded to the adsorbent surface, effectively filling its initially porous structure. This leads to a smoother surface, and the darker areas observed confirm that phenol has covered the carbon surface, demonstrating successful adsorption of phenol onto the adsorbent. Furthermore, the decrease in pore visibility suggests the presence of adsorbate layers, suggesting that phenol molecules have taken up the available adsorption sites in the pore spaces.

The supplementary file Fig. S2 shows a study of Energy-dispersive X-ray spectroscopy (EDS). Several significant elements were found on the surface of the activated carbon, according to the EDS analysis of BTPAC. The percentages of carbon (C), sodium (Na), silicon (Si), oxygen (O), and phosphorus (P) that were found during the pre- and post-adsorption procedures are among these components. In the pre-adsorption data, the high carbon content of 78.23% confirms the effective conversion of agricultural waste into activated carbon. A high carbon percentage indicates that a highly porous carbonaceous structure has been generated after the majority of the volatile components were eliminated during the preparation and activation process. Oxygen (9.63%) is the second-highest proportion in the EDS examination, confirming the existence of functional groups. This comprises carbonyl (–C=O), hydroxyl (–OH) and carboxyl (–COOH). During the activation process, partial oxidation is indicated by the presence of oxygen, which makes it easier to introduce active sites for adsorption. The acidic treatment of the activated carbon corresponds to the phosphorus level (5.02%)^71^. Before adsorption, the material exhibited a rich-carbon content of 78.23% and a relatively lack of oxygen content of 9.63%, indicating successful carbonization. In the post-adsorption EDS analysis, the carbon content reduced by 3.05%, while the oxygen percentage raised by 1.08%, which implies that BTPAC and the phenol pollutant interact. Additionally, the percentage of phosphorus (P) was higher after adsorption compared to before. These observations support previously reported studies^72^.

### Point of zero charge (pHpzc) determination of BTPAC

**Fig. 4:**
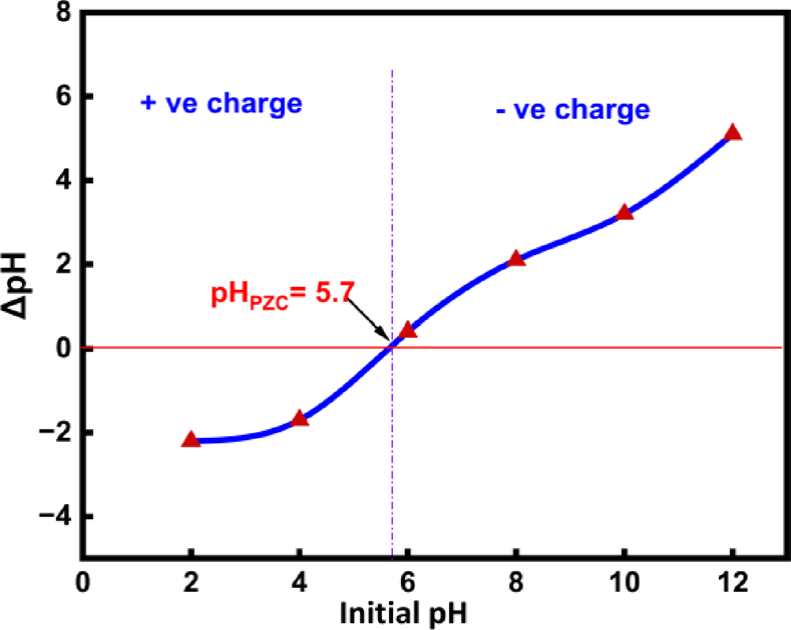
Determination of pHpzc of BTPAC.

The surface characteristics were determined by the pHpzc. Figure 4 demonstrates that the pHpzc of BTPAC is 5.7, indicating that chemical activation enhances the formation of oxygenated surface groups that have an acidic nature on the activated carbon. According to the figure, the material’s surface has more positive charges when the pH solution is less than pHpzc, and more negative charges when the pH is greater^73^.

### Boehm titration determination of activated carbon

Boehm titration results (Supplementary file: Table S3) confirmed the main oxygen-comprising functional groups on the activated carbon surface were carboxylic (0.389 meq/g), phenolic (0.81 meq/g) and lactonic (0.12 meq/g) groups. It was discovered that the overall acidic group content was 1.317 meq/g, in contrast to the total basic group content of 0.038 meq/g. This suggests that the existence of acidic groups on the surface is noticeably greater. Therefore, it confirms that the produced adsorbent has a predominantly acidic composition^74^. The observed pHpzc value and the Boehm titration findings agreed well, confirming the activated carbon surface’s acidic nature^36^.

### FTIR spectra analysis of BTPAC

**Fig. 5:**
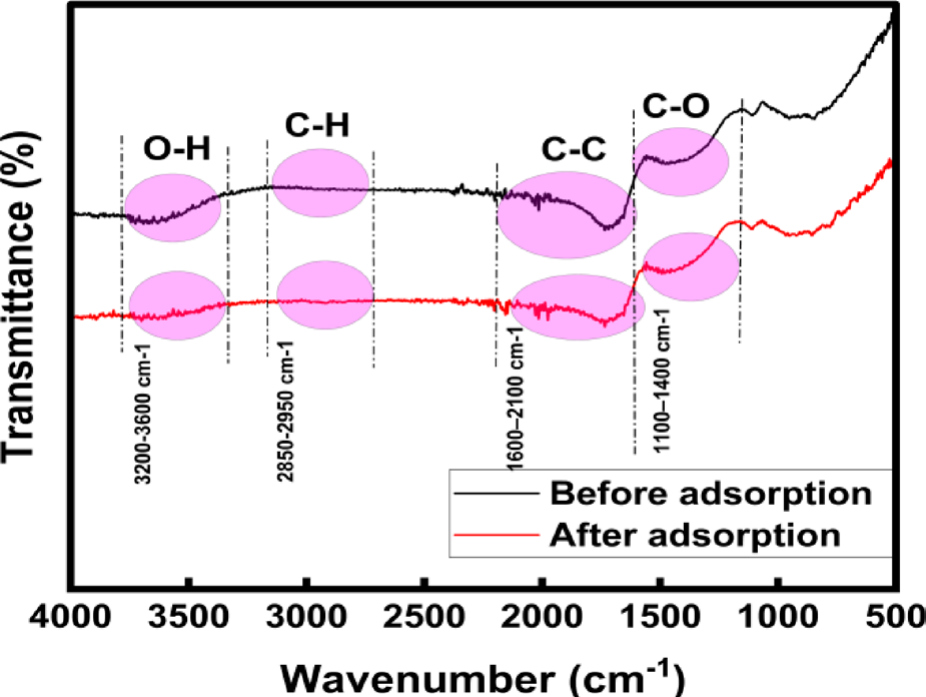
FTIR analysis of BTPAC—pre and post adsorption.

The FTIR spectra analysis, which is shown in Fig. 5, reveals distinctive changes in peak positions and variations in peak intensities, which offer crucial insights into the adsorption mechanism.

The broad band seen in the pre-adsorption data, which spans 3200–3600 cm^−1^, is indicative of the O–H bond stretching of the hydroxyl groups and water molecules on the surface. The region between 2850 and 2950 cm^−1^ indicates C–H stretching modes. Additionally, the C=O stretching of carbonyl, carboxyl, or lactone functional groups is responsible for a noticeable peak close to 1700 cm^−1^^75^. Peaks at 1700–1500 cm^−1^, related to C=C bond stretching, which is related to the aromatic rings, confirming the graphitic structure of activated carbon. Peaks at 1700–1500 cm^−1^, allied with C=C stretching of aromatic rings, confirming the graphitic nature of activated carbon. Additionally, the 1200–1000 cm^−1^ section displays peaks related to C–O bond stretching vibrations from oxygen-present groups, such as phenolic, ether, or lactone^29^. After analyzing the post-adsorption data, the O–H bond stretching shows a reduction in intensity, which suggests hydrogen bonding between phenol molecules and surface hydroxyl groups. Additionally, the C=O peak displays a minor shift and a reduction in intensity, indicating interactions among the adsorbent surface and functional groups of phenol pollutant^76^. The adsorption of phenol molecules is shown by new peaks at 1500–1400 cm^−1^, which are allied with C–C bonding in the aromatic rings. Notable alterations in C-O stretching area validate the role of oxygenated functional groups in adsorption^45^. Hydrogen bonding is indicated by the interaction between the hydroxyl (–OH) groups of phenol and the oxygen-containing surface groups of activated carbon^75^. The π–π stacking occurs among the aromatic rings of phenol and the graphitic domains of activated carbon. Furthermore, electrostatic attractions may occur depending on the surface charge of activated carbon under the optimal pH conditions. These highlight the behaviour of adsorption on BTPAC, involving both physical and chemical interactions.

### XPS analysis of BTPAC

**Fig. 6:**
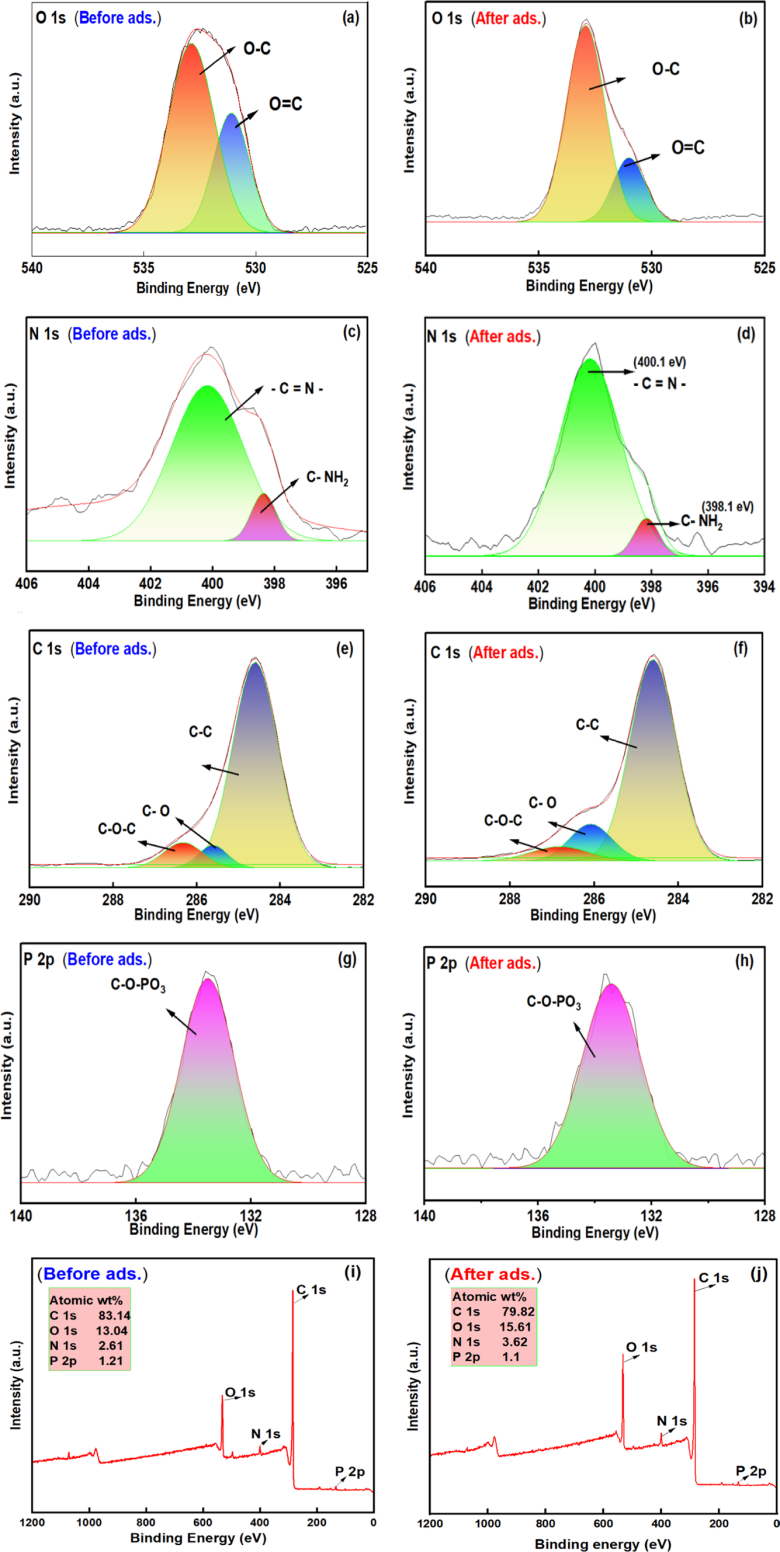
XPS Analysis of BTPAC- before and after phenol adsorption.

The XPS analysis of BTPAC, both pre- and post-phenol adsorption, is shown in Fig. 6a–j, which reveals the peaks of different elements and provides the atomic ratio of BTPAC.

According to the study, the main elements found in BTPAC are carbon (C 1s), oxygen (O 1s), phosphorus (P 2p), and nitrogen (N 1s). Two peaks were visible in the O 1s X-ray photoelectron spectroscopy (XPS) spectrum: one at 531.4 eV, which is associated with carbonyl groups (O=C), and another at 533.7 eV, which is associated with C–OH or O–C groups. The slight shift in binding energy towards lower values after adsorption indicates that oxygen-comprising functional groups may be involved in the adsorption mechanism, likely involving hydrogen bonding or π–π assembling with phenol molecules^77^. In the N1s XPS spectrum, two distinct peaks were observed at approximately 398.5 eV and between 400.0 and 400.5 eV. The peak at 398.5 eV corresponds to terminal amine groups (C-NH₂), which provide active sites for phenol adsorption. The peak in the range of 400.0 to 400.5 eV is associated with carbon-conjugated nitrogen species (C=N), which enhance the adsorption and electron-donating capabilities of BTPAC^78^. Additionally, three peaks at various binding energies of 284.6, 285.5, and 286.9 eV were seen in the C1s XPS spectra; these peaks corresponded to C–C, C–O, and C–O–C bonds, respectively^79^. The C–O bond is formed in part by the existence of functional groups such carboxylic, aldehydes, ketones, and aliphatic ethers. Significant alterations were seen in the deconvoluted C1s spectra of activated carbon before to and following phenol adsorption. Significantly, the C-O and O-C = O peak existence has increased, suggesting the participation of functional groups that contain oxygen. Furthermore, π–π interactions among phenol and the carbon surface are suggested by the shift in the C–C peak towards lower binding energy. These results demonstrate that both chemical interactions which is hydrogen bonding and physical bindings may be π–π interactions regulate the phenol adsorption process. The P 2p XPS spectrum displayed a strong peak at 134.1 eV, characteristic of pentavalent phosphorus species. This corresponds to phosphate groups (P^5^⁺) associated with bonding environments like C-O-PO_3_ and P-O-C^80^. The effective adsorption of phenolic molecules onto the activated carbon was evidenced by a decrease in carbon content to 79.82% and an increase in oxygen content to 15.61% after phenol adsorption. This rise in oxygen percentage can be ascribed to the contact between adsorbate groups and the activated carbon, which happens over hydrogen bonding and π–π interactions. Additionally, a slight decrease in nitrogen and phosphorus levels was observed, likely due to surface coverage following adsorption, as illustrated in Fig. 6i,j.

### TGA analysis of BTPAC

**Fig. 7:**
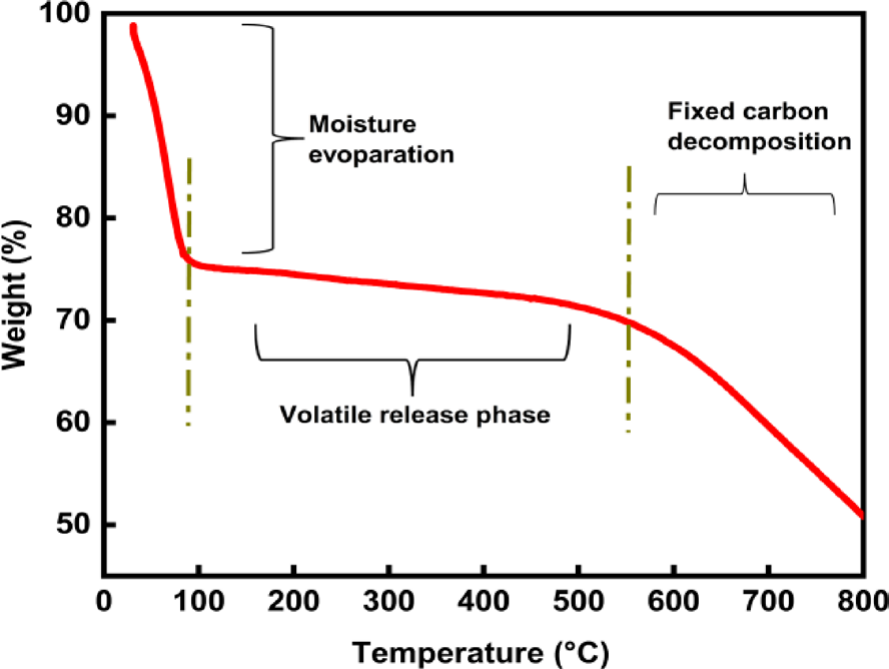
TGA Analysis of BTPAC.

The thermogravimetric analysis (TGA) of BTPAC is shown in Fig. 7. It has a comprehensive detail regarding its thermal weight decrease data. The initial stage of the experiment shows a gradual decline in weight percentage, decreasing from 100% to approximately 75–80%. This drop is primarily attributed to the loss of surface and bound moisture within the activated carbon derived from bauhinia tree pods.

During this initial phase, which occurs between 100 and 120 °C, there is physical adsorption of water molecules, and the weight reduction is minimal in comparison to the later thermal phase of the adsorbent. As the temperature increases, weight loss becomes more pronounced, indicating the breakdown of leftover organic molecules within the activated carbon. After reaching 150 °C, the rate of weight loss begins to slow down; however, a gradual decline in mass continues to be observed between 200 and 600 °C, resulting in a sustained weight loss of 5% to 10%. During this phase, the decomposition of residual organic compounds takes place: hemicellulose-like residues break down between 200 and 300 °C, while any remaining cellulose thermally decomposes between 300 and 400 °C^81^. Meanwhile, lignin gradually breaks down across a wide thermal range of 200–600 °C, which aids in long-term weight loss^82^. Above 600 °C, the final phase shows a consistent weight reduction, most likely caused by the gradual decomposition of thermally stable functional groups and the slight volatilization of carbonaceous materials. This phase results in an additional weight loss of 15 to 20%. This stage represents the carbonization process, which increases the material’s fixed carbon content by removing non-carbon components, including oxygen and hydrogen. The ash content, consisting of inorganic minerals and contaminants that do not volatilize at high temperatures, is reflected in the residual weight above 800 °C.

### Adsorption experimental studies

Adsorption experiments were conducted on BTPAC at the optimum adsorption parameters: equilibrium time of 210 min, initial concentration phenol pollutant of 30 mg/L, BTPAC’s dosage of 1.6 g/L, pH of 4, temperature of 25 °C, and agitation speed of 150 rpm. The effect of equilibrium time and other parameters is discussed below.

## Influence of optimisation parameters

### Influence of equilibrium time for adsorption of phenol on BTPAC

**Fig. 8:**
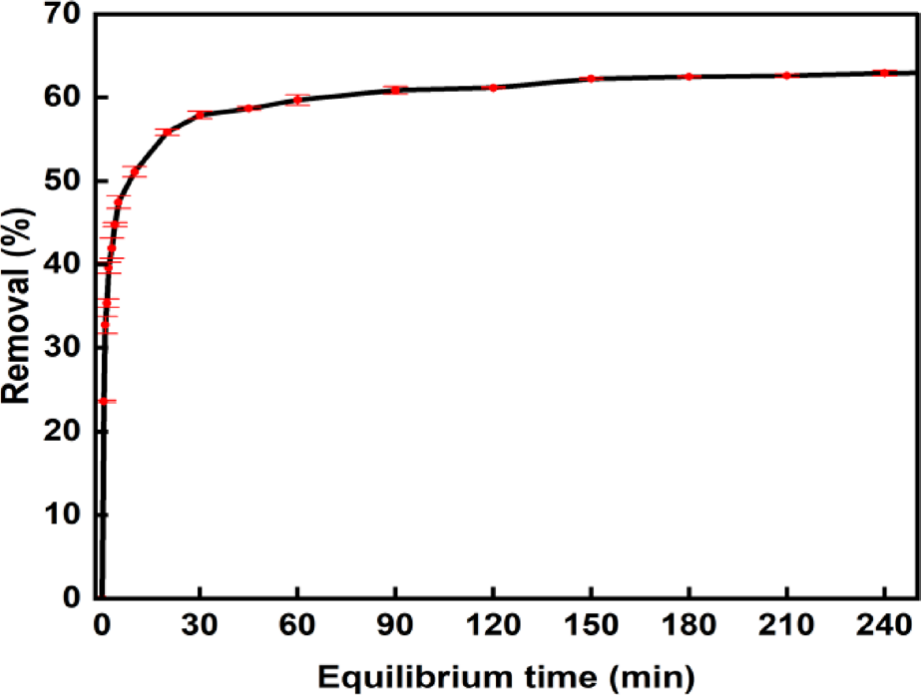
Influence of equilibrium time for adsorption of phenol on BTPAC (Exp. conditions: Dosage, 1 g/L; pH, 7; concentration, 30 mg/L; Speed, 120 rpm).

The results of the equilibrium time investigation are illustrated in Fig. 8. This graph explores that the efficacy of phenol removal was significantly increased in the early stages of the adsorption behaviour. The substantial concentration differential between the adsorbent and the phenol solution is primarily responsible for the initial significant increase in removal efficiency. This difference creates a strong driving force for molecular movement toward the adsorbent. Furthermore, the BTPAC’s surface has a huge number of accessible active sites at the start of the process, which promotes the solute’s quick and effective adsorption. The removal efficiency quickly escalates, reaching 50% within the first 20 min, and achieves its peak value before 210 minutes^83^. As most active sites become occupied, extending the contact time further does not enhance adsorption. From both operational and financial standpoints, prolonging the adsorption period unnecessarily inflates energy and processing costs without providing additional benefits. These findings resulted in the selection of 210 min as the optimal contact time for this investigation^75^.

#### Influence of dosage, pH, temperature and initial concentration

Using Response surface methodology (RSM) approach, the effects of four critical parameters, temperature, absorbent dosage, pH, and initial phenol pollutant concentration were examined to ascertain their impact on phenol removal. The Design-Expert ANOVA application suggested a quadratic model^84^. The experimental design framework, along with the predicted and actual phenol removal percentages, is presented in the supplementary file Table S4.

The Eq. (13) shows the empirical models that relate to the removal of phenol in the form of coded factors:

13 $$\begin{aligned} R1 & = + 53.56 + 18.2A - 1.54B - 5.46C - 7.53D \\ & \quad - 0.1188AB - 0.6438AC - 2.07AD - 0.22BC \\ & \quad - 0.245BD - 0.1425CD - 4.75A^{2} + 1.34B^{2} + 2C^{2} - 2.52D^{2} \\ \end{aligned}$$

**Fig. 9:**
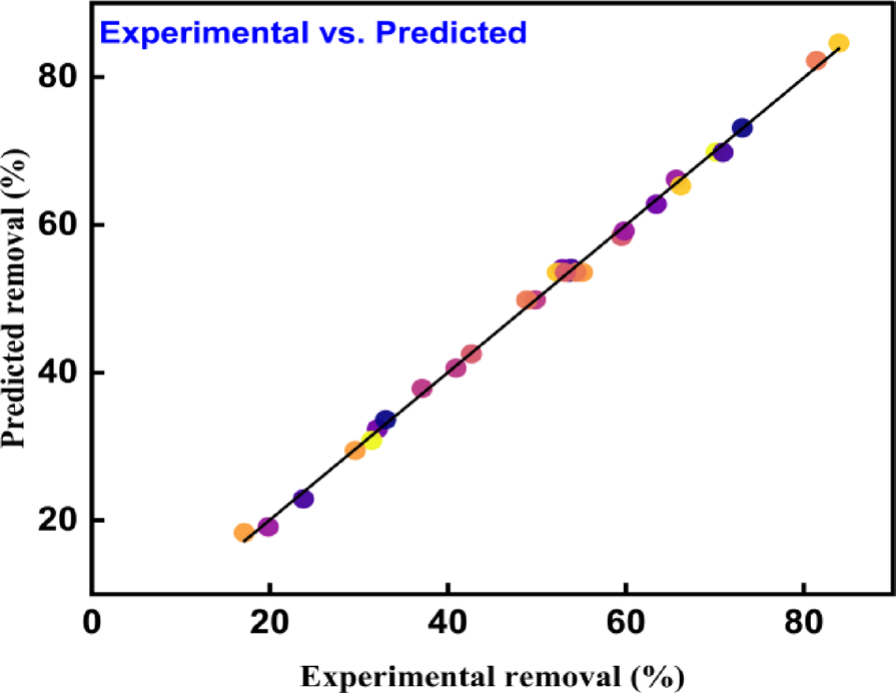
The graph plot of experimental vs predicted phenol removal percentage as per RSM.

The phenol removal percentage is denoted by $R1$, the dosage by $A$, the pH by $B$, the temperature by $C$, the initial concentration by $A$, and the interaction effects of the corresponding optimisation factors. The model’s relevance was determined using the values of P and F. The significance of the model data is demonstrated by the F value of 553.56 and the *p* value of less than 0.0001. These numbers show a high correlation between the chosen optimisation parameters and the response factor (elimination percentage). *p* value < 0.05 indicate that a model term is significant^76^. Additionally, with the largest value of F is 5834.01 and a *p* value of less than 0.0001, the adsorbent dosage is the most significant parameter. Table 3 provides the ANOVA model values from RSM approach of phenol adsorption for the quadratic model. Figure 9 displays the expected vs. actual graph plot for phenol removal percentage according to RSM.

**Table 3:** ANOVA model values from RSM approach of phenol adsorption.

Factors	SSE	DF	Mean Square	F value	*p* value
Model	8797.86	14	628.42	553.56	< 0.0001*
A-Dosage	6622.91	1	6622.91	5834.01	< 0.0001*
B-pH	47.59	1	47.59	41.92	< 0.0001
C-Temperature	595.86	1	595.86	524.88	< 0.0001*
D-Concentration	1135.48	1	1135.48	1000.22	< 0.0001*
AB	0.2256	1	0.2256	0.1987	0.6621
AC	6.63	1	6.63	5.84	0.0289
AD	68.48	1	68.48	60.32	< 0.0001
BC	0.7744	1	0.7744	0.6822	0.4218
BD	0.9604	1	0.9604	0.846	0.3722
CD	0.3249	1	0.3249	0.2862	0.6005
A^2^	210.2	1	210.2	185.16	< 0.0001*
B^2^	16.87	1	16.87	14.86	0.0016
C^2^	37.31	1	37.31	32.86	< 0.0001
D^2^	59.42	1	59.42	52.34	< 0.0001
Residual	17.03	15	1.14		
Lack of Fit	12.06	10	1.21	1.21	0.4394
Pure Error	4.96	5	0.9929		
Cor Total	8814.88	29			

*The factors are significant.

According to ANOVA statistics, since the difference between the expected R^2^ is 0.9920 and the adjusted R^2^ value of 0.9963 is less than 0.2, proposed model is in good correspondence.

### Influence of individual and the interactive effect

The ANOVA-generated graph of one factor and the interaction effect of contour plots is shown in the supplementary file Fig. S3.

#### Influence of varying adsorbent dosage on phenol pollutant on BTPAC

The dosage effect plot illustrates that the percentage removal effectiveness of the adsorbate rises with the adsorbent dosage. The availability of increased adsorption sites enhances the adsorption process and improves removal efficiency. This is evident from the upward trend in removal percentage values as the dosage increases. Experimental findings indicate that an optimal dosage of 1.6 g/L achieves a balance between high removal efficiency, adequate adsorption capacity, and economic feasibility. Using more adsorbent beyond this dosage is not cost-effective, as the improvement in removal efficiency is only marginal. Additionally, using excessive adsorbent could result in particle clumping, which reduces the available surface area of BTPAC and diminishes adsorption efficiency. Therefore, in this study, 1.6 g/L is taken as the ideal dosage to maintain high efficiency while minimizing costs in practical applications.

#### Effect of solution pH on phenol pollutant on BTPAC

According to the ANOVA plots, the adsorbate is removed quicker at lower pH. This behaviour is explained by the fact that the adsorbent’s surface converts positively charged at lower pH values, which increases the adsorption of negatively charged adsorbate ions by electrostatic attraction. Excess H^+^ ions and phenol ions competed for the accessible sites on BTPAC’s at an acidic pH, however at a higher pH, there is less competition from H^+^ ions, which results in a reduced removal percentage^85^. Based on RSM analysis, the lower pH value of 4 is considered as optimum pH.

#### Influence of adsorbent with temperature on BTPAC-induced phenol adsorption

In terms of temperature effect, there was clear evidence that lower temperatures promoted a higher proportion of phenol removal as per the ANOVA data. As the temperature rises, the bond strength or attractive forces between BTPAC and the phenolic compounds may reduce, which could explain this reduction. The spontaneous tendency and exothermic character of phenol adsorption on BTPAC can be seen by the decline in removal effectiveness with an increase in operating temperature^64^. Other researchers have also noted identical results for phenol adsorption on activated carbon^86^. Based on the response surface methodology analysis, the lower temperature of 25 °C is decided as optimum.

#### Influence of initial concentration effect of adsorption of phenol on BTPAC

As the initial concentration increases from 20 to 40 mg/L, the removal percentage slightly decreases. The removal effectiveness decreases as its initial concentration increases. This decrease is explained by surface saturation caused by the restricted number of active adsorption sites at higher phenol concentrations^41^. Depending on the experimental data, 30 mg/L was identified as the optimum concentration, balancing high removal efficiency, industrial relevance, and economic feasibility. At 30 mg/L, the adsorption process achieves an optimal removal efficiency. Additionally, this concentration aligns with industrial wastewater standards, as many industries discharge wastewater containing pollutant concentrations around 20–30 mg/L, making this investigation relevant for real-world applications. From an economic perspective, treating higher concentrations requires more adsorbent material, increasing operational costs and making the process less viable for large-scale implementation. The adsorption efficiency of BTPAC rises with an increase in initial concentration, while the removal efficiency decreases when the active sites of the adsorbent become saturated^87^.

#### Interactive effect of adsorption variables

The darker areas in the contour map show how dosage and temperature interact to affect phenol adsorption by activated carbon as shown in Fig. S3 (Supplementary file), showing that removal efficiency rises with increased adsorbent dosage and lower temperatures. The impact of dosage is more on the removal efficiency compared to its change in temperature. The way that temperature and pH interact to affect the removal of phenol by activated carbon shows that removal efficiency increases at lower temperatures, especially at lower pH levels. This implies that the adsorption process might be exothermic, in which phenol absorption is favoured by lower temperatures because they reduce molecule agitation and improve contact with active sites. Lesser pH causes the surface of activated carbon to convert more protonated, which improves phenol adsorption through π–π interactions and hydrogen bonds, particularly when phenol is still in its molecular form. Thus, a combination of a lower temperature and an acidic pH greatly improves adsorption efficiency. Significant interactive effects between dosage and pH adsorption parameters indicate that the higher dosages and lower pH values result in increased phenol removal effectiveness. Furthermore, the interaction among the initial concentration and dosage of adsorbent on activated carbon adsorption demonstrates that removal efficiency increased with lower phenol concentration and higher dosage. Adsorption is more successful at reduced concentrations because there are more accessible active sites relative to phenol molecules.

### Adsorption isotherm studies of adsorption of phenol on BTPAC

**Fig. 10:**
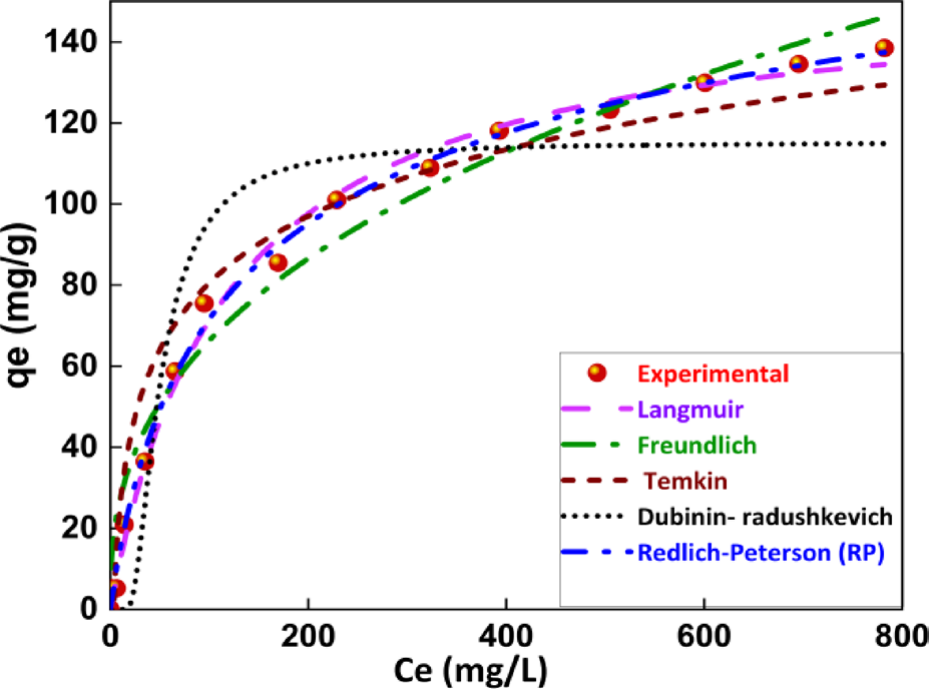
Adsorption isotherm experimental curves with model curves.

The nonlinear adsorption isotherm modelling for adsorption of phenol on BTPAC is investigated from increasing concentration 10 to 1000 mg/L as shown in Fig. 10 and the corresponding parameter values are shown in Table 4. This study investigated the adsorption behaviour of phenol onto BTPAC to comprehensively understand the adsorption mechanisms and surface interactions by fitting different isotherm model and parameters. According to the Langmuir isotherm model, adsorbed molecules do not move laterally on the surface, and each adsorption site has the same sorption activation energy. The energy constant related with the heat of adsorption is identified as the Langmuir parameter $({K}_{L}$)^88^. Stronger binding (greater affinity) among the adsorbate and the surface of activated carbon is indicated by a higher ${K}_{L}$ value. Freundlich isotherm describes adsorption on the heterogeneous surface, where many layers of adsorption may occur. The Freundlich constant (${K}_{F}$) can be represented as a distribution coefficient, indicating how much phenol is absorbed onto a unit of activated carbon at equilibrium. The parameter *n* specifies the degree of non-linearity amongst the adsorbate’s concentration in solution and its adsorption onto a surface. It shows the adsorption process’s mechanics and favourability. The Temkin isotherm accounts for interactions between the adsorbent and the adsorbate, the heat of adsorption decreases as coverage increases. The Temkin parameters such as ${A}_{T}$ and ${B}_{T}$ are linked with binding energy and heat of adsorption respectively. At low sorbate concentrations, the Redlich-Peterson (RP) isotherm model follows Henry’s rule, but at higher concentrations, it displays qualities of the Freundlich model. The RP model parameter $B$ equals to 1, it behaves like the Langmuir isotherm; when $\text{B}$ equals 0, it follows the Freundlich isotherm^89^. The Dubinin-Radushkevitch (DR) model parameters, like $\beta r$ and ε represent the strength of adsorption and whether it is physical or chemical. As indicated by Table 4, The higher ${R}^{2}$ values, along with the lower ${\upchi }^{2}$ values and sum of squared errors (SSE), suggest that the Langmuir model fits the experimental data very well. This implies that monolayer adsorption of phenol molecules results from the adsorption mechanism mainly occurring on particular active sites of the BTPAC surface. It suggests a homogeneous distribution of adsorption sites with finite adsorption capacity. These results emphasize the importance of particular binding interactions among the adsorbate and the activated carbon’s surface and validate that phenol adsorption over BTPAC follows a well-defined and organised adsorption pattern. Even the $B$ value of 0.88 from the RP model implies that the experimental value fits the Langmuir model better, with a value near 1. A significant improvement in the adsorption process’s favourability was indicated by the phenol adsorption separation factor (${R}_{L}$)^76^, which dropped from 0.97 to 0.26 which shown in the supplementary file Fig. S4. Considering the ${R}^{2}$ value, SSE and ${\upchi }^{2}$ value, the sequence of fit was Langmuir > RP model > Freundlich > Temkin isotherm > DR model concerning phenol.

**Table 4:** An overview of the isotherm parameters for the phenol adsorption on BTPAC.

Isotherm models	Constants	Values	Units	*R*^2^	${\upchi }^{2}$	SSE
Langmuir isotherm	${q}_{m}$ ${K}_{L}$	185.93 0.00291	mg/g L/mg	0.99	5.23	68.0
Freundlich isotherm	${K}_{F}$ *n*	11.33 0.38	((mg/g)/(L/mg)^1/n^ Constant	0.97	65.92	791.1
Temkin model	${B}_{T}$ ${A}_{T}$	23.76 0.29	J/mol L/mol	0.93	164.1	1969.3
Dubinin-Radushkevich model	${q}_{m}$ $\beta r$ ε	115.27 1.4E-06 707	mg/g Constant J/mol	0.74	145.52	14,498
Redlich-Peterson model	${K}_{RP}$ ${A}_{RP}$ ${\rm B}$	1.78 0.02 0.88	L/g L/g Constant	0.99	8.27	99.2

### Adsorption kinetics of phenol on BTPAC

The rate constant obtained from the kinetics study indicates the speed at which adsorption attains equilibrium^90^. Fig. S5 (supplemental file) shows the plot of adsorption kinetics of phenol pollutant’s on BTPAC in this investigation. It is preferable to fit a nonlinear model in order to improve accuracy and deal with time-based dependence’s limitations. Pseudo first-order (PFO), Pseudo-second-order (PSO), intraparticle diffusion (IPD), and Elovich models are used to fit with experimental data. In adsorption kinetics, the rate constants of PFO and PSO kinetic models are denoted by ${k}_{1}$ and ${k}_{2}$, respectively. Assuming that the rate is dependent on unoccupied surface sites, ${k}_{1}$ is related to physical adsorption. Although it might not work well for complex systems, a higher ${k}_{1}$ indicates faster initial adsorption, assuming that the chemical interactions among the activated carbon and the adsorbate control the rate, ${k}_{2}$ which is associated with chemisorption. Quicker chemical bonding is indicated by a higher ${k}_{2}$. The majority of the time, ${k}_{2}$ gives a better fit to experimental data, particularly when chemisorption is dominant. The IPD model parameter shows how quickly the adsorbate diffuses through the pores of the adsorbent, ${K}_{diff}$. Additionally, the adsorbent particle’s boundary layer thickness is correlated with *C* (intercept). The Elovich kinetic model parameters *(a, b)* are related to initial rate of adsorption capacity and ease of desorption. The PFO models *R*^2^ values are in between 0.93 and 0.97, suggesting a reasonable but not perfect fit to the experimental values. The result revealed that the phenol adsorption on BTPAC is good fitted with the PSO model, with a higher *R*^2^ value of 0.99 as compared to other kinetic models, which is very close to 1. The phenomenon indicates that the PSO rate constant decreases as $Co$ increases, and the experimental values align perfectly with the theoretical values, as shown in supplementary file table S5. The ${\upchi }^{2}$ (Chi-square) shows lower deviations for the PSO model, confirming its superior fit compared to the other models. The study reveals that the adsorption rate depends on the square of the available adsorption sites, making it more suitable for chemisorption processes. This model takes into consideration interactions in which the phenol pollutant and the BTPAC share or exchange electrons to achieve adsorption. Similar data’s are reported by previous research papers^33^. The adsorption kinetic model parameters for phenol adsorption onto BTPAC is shown in Table S5 (Supplementary file).

### Activation energy for the adsorption of phenol

**Fig. 11:**
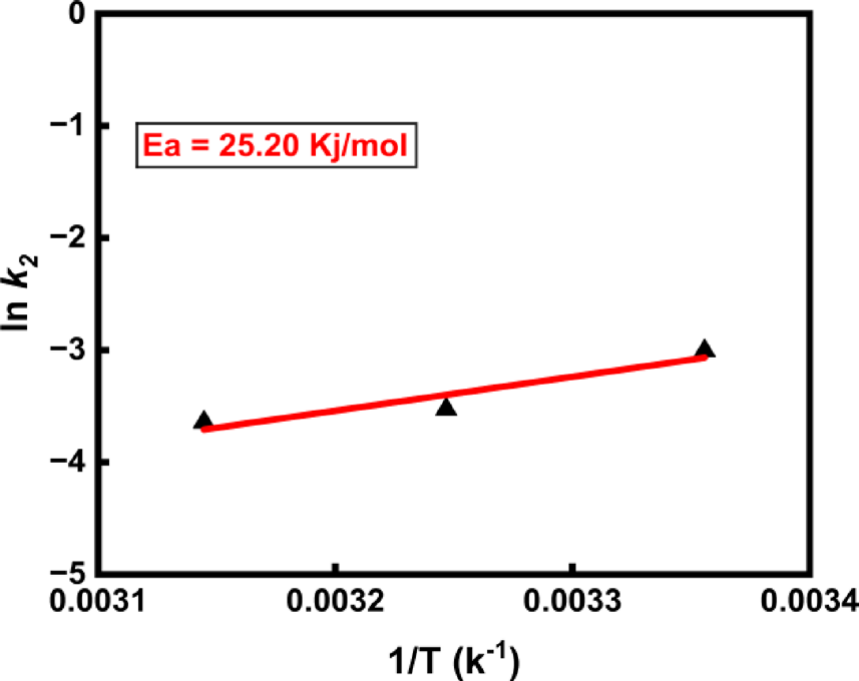
Determination of the activation energy for Phenol.

In this study, the activation energy ($Ea$) for the adsorption of phenol was assessed to be 25.20 kJ/mol. The details of the graph of varying temperature vs kinetics are given in Fig. 11. It shows that the necessary activation energy is within the usual range for physical adsorption, these findings imply that physical adsorption dominates the adsorption process.

Physical adsorption processes are quickly reversible and reach equilibrium easily. They typically require lower activation energies, usually between 5 and 40 kJ/mol. In contrast, chemical adsorption features significant interactions and requires more substantial activation energies, varying from 40 to 800 kJ/mol^91^. The type of binding forces involved, stronger covalent or ionic bonds in chemical adsorption versus weaker van der Waals forces in physical adsorption, causes the variation in energy requirements. These qualities are essential in applications involving adsorption-based water treatment. Physical adsorption is a relatively low-energy technique that is effective at lesser temperatures owing the weak van der Waals forces involved. The lower activation energy values observed in this study support the idea that phenol adsorption occurs through a physical adsorption pathway, allowing it to reach equilibrium more quickly than chemical adsorption. This distinction is crucial for optimizing water treatment processes that rely on adsorption, as it influences factors such as temperature control, energy consumption, and the selection of suitable adsorbents.

### Thermodynamics studies for the adsorption of the phenol on to BTPAC

**Fig. 12:**
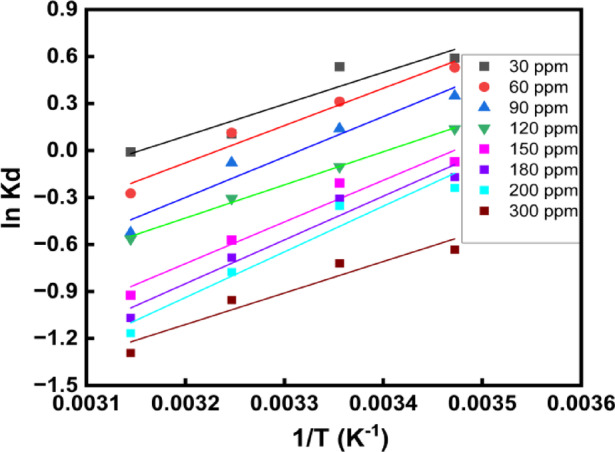
van’t Hoff plot for adsorption of phenol on BTPAC.

To more comprehensively know the adsorption behaviour and feasibility, the thermodynamic behaviour of phenol adsorption onto BTPAC was methodically examined across a variety of temperatures. As shown in Fig. 12, the variation of *(*$1/T$*)* versus *(*$ln{k}_{d}$*)* corresponds to a recognised thermodynamic method for assessing adsorption properties. The fundamental thermodynamic variables are largely determined by the relationship of Gibbs free energy (∆G^o^), Enthalpy change (∆H^o^), and Entropy change (∆S^o^) which gives important knowledge into the spontaneity, energy dynamics, and disorder of the adsorption system. These parameters are presented in Table 5.

**Table 5:** Thermodynamic adsorption parameters for phenol adsorption onto BTPAC.

Adsorbate	C_o_ (mg/L)	∆G^o^ kJ/mol				∆H^o^	∆S^o^
		289 K	299 K	309 K	319 K	kJ/mol	kJ/mol/K
Phenol	30	− 2.48	− 2.45	− 1.41	− 1.14	− 17.07	− 0.05

The negative ΔG° values at every temperature shows that the phenol adsorption is spontaneous. Conversely, the amount of ΔG° decreases as the temperature rises, indicating that spontaneity decreases at higher temperatures. This implies that lower temperatures favour phenol adsorption, consistent with the exothermic behaviour. The calculated ΔH° value is -17.07 kJ/mol, illustrating that the adsorption nature is exothermic. Usually, the Van der Waals interactions or hydrogen bonding are frequently linked to exothermic adsorption, which facilitates physical adsorption. The value of ΔS° is − 0.05 kJ/mol K, suggesting a decrease in randomness at the BTPAC to phenol solution interface during adsorption. This negative entropy change indicates that the phenol molecules become more ordered on the adsorbent surface upon adsorption. When phenol molecules are adsorbed from the solution phase onto the solid surface, a negative ΔS° value has been interpreted as their loss of freedom. Information regarding various agricultural sources and their phenol adsorption capacities is included in Table 6. The BTPAC is showing an evident performance compare to other sources and stand out as one of the choices for treating phenolic contaminants.

**Table 6:** Comparison of adsorption capacity and preparation conditions for phenol adsorption with other adsorbent sources.

Adsorbent source	Experimental conditions				${q}_{m}$ (mg/g)	References
	Dosage (g/L)	Contact time (min)	C_o_ (mg/L)	Temp. (°C)		
Pine fruit shells	0.5	1440	20–100	25	26.7	^92^
Palm kernel	0.6	50	10–100	25	10.02	^93^
Rice husk	2	180	5–20	35	13.98	^94^
Brazil nut shells	0.75	180	25–200	55	99	^95^
Wood apple shell	0.8	150	100–400	30	102.71	^96^
Sugarcane bagasse	0.5	60	50–250	25	158.9	^27^
*Bauhinia monandra* pods	1.6	210	10–1000	25	185.93	This work

### Isosteric heat of adsorption

**Fig. 13:**
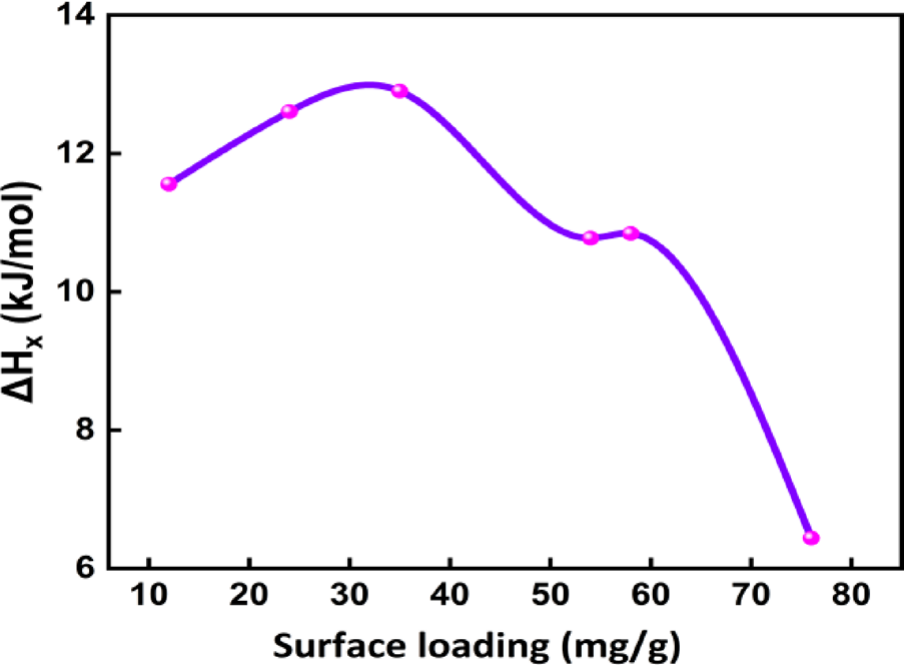
Isosteric heat of adsorption curve.

The Clausius Clapeyron equation was used to calculate the isosteric heat of adsorption $(\Delta Hx$). The calculation was based on equilibrium concentration data obtained at four different temperatures for fixed amounts of adsorbed phenol. For each fixed equilibrium loading (${q}_{e}$), the corresponding equilibrium concentrations (${C}_{e}$) were extracted from isotherms plotted at 289 K, 299 K, 309 K, and 319 K. The calculated $\Delta Hx$ values were then plotted against the corresponding surface loadings to understand how adsorption energy varies with surface coverage. As shown in Fig. 13, $\Delta Hx$ exhibited a decreasing trend with increasing ${\text{q}}_{\text{e}}$, indicating that high-energy adsorption sites are occupied at low surface coverage, while lower energy sites become active as coverage increases. When adsorption initially takes place at stronger binding sites, this behaviour is typical of a heterogeneous adsorbent surface. For all coverages, the value of $\Delta Hx$ was less than 25 kJ/mol, which strongly implies that physisorption mechanisms dominated the adsorption of phenol onto BTPAC. These values align with previously documented ranges for physical adsorption, they usually require π–π interactions, van der Waals forces, or weak hydrogen bonds among the phenol solution and the aromatic surface of the BTPAC.

### Desorption and regeneration of BTPAC

**Fig. 14:**
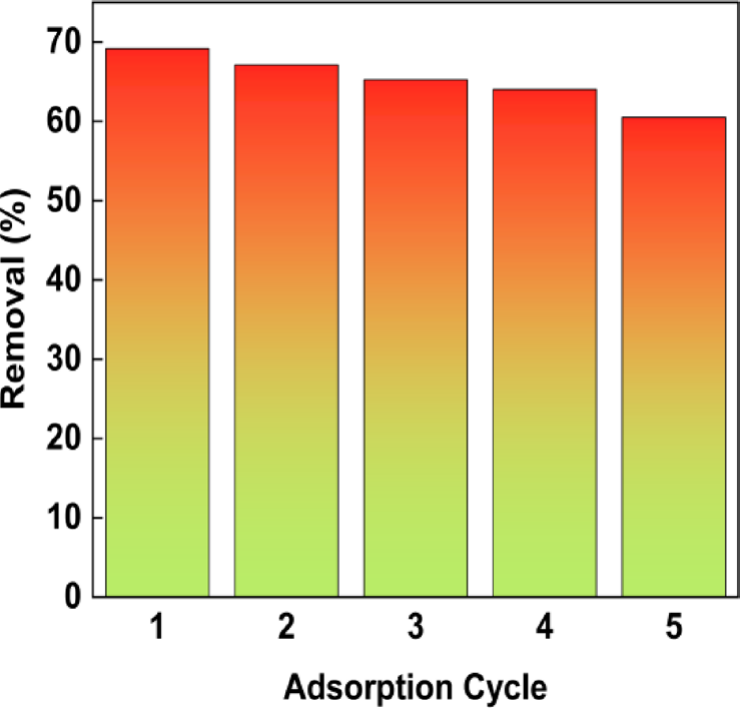
Desorption study of BTPAC on phenol.

The removal efficiency of the adsorbent remained relatively constant over three to four consecutive adsorption–desorption cycles, as illustrated in Fig. 14. However, a noticeable decline occurred in the fifth cycle, with removal effectiveness decreasing from 69% in the first cycle to approximately 64% in the fifth. This drop may indicate either the gradual saturation of adsorption sites or changes in the adsorbent due to frequent use. Despite this slight decrease, the overall performance suggests that the adsorbent maintains its effectiveness for several cycles before a significant decline in efficiency is observed. BTPAC indicates it has the capability or potential to be used in wastewater treatment in the real world through its consistent performance across multiple adsorption–desorption cycles. Its high desorption efficiency and minimal loss of adsorption capacity make it a viable option for environmentally friendly and cost-effective pollutant removal.

### Performance of activated carbon in eliminating phenol from aqueous systems (Spiked Study)

**Fig. 15:**
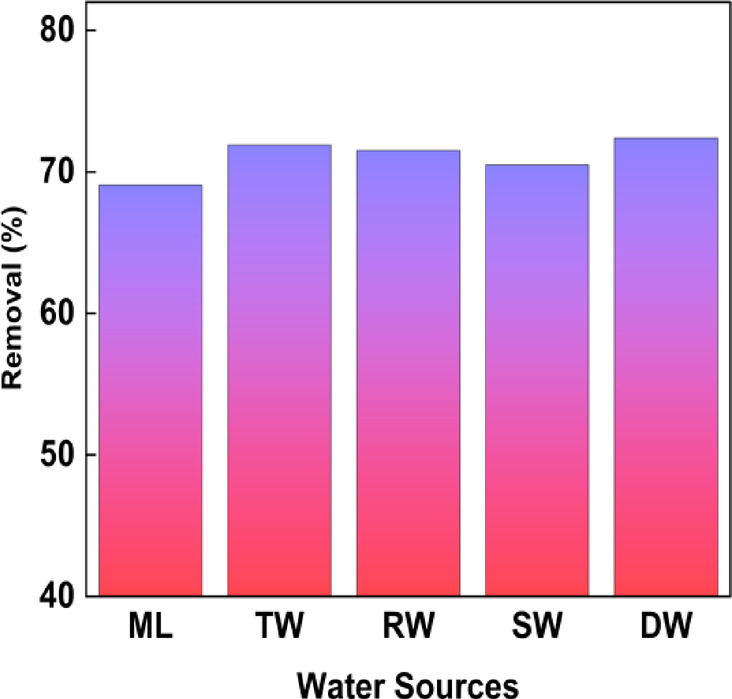
Phenol removal efficiencies across different water sources from Manipal lake (ML), tap water (TP), river water (RW), sea water (SW), and distilled water (DW).

The phenol removal efficiency for different water sources is displayed in Fig. 15. To evaluate the practical applicability of the synthesised porous material, the adsorption tests were executed on actual water samples that had been purposefully spiked with known quantities of phenol. To ensure the samples reflected typical environmental conditions, grab samples were collected from a range of natural water sources, including tap water, Manipal Lake, river water, distilled water, and seawater. After proper filtration, adsorption was performed under optimized laboratory conditions. Even though the collected samples contained organic matter and background ions, the results showed significant phenol removal efficiency. This confirms the effectiveness of BTPAC beyond synthetic solutions, highlighting its resilience and adaptability in handling diverse water matrices.

### Mechanism

**Fig. 16:**
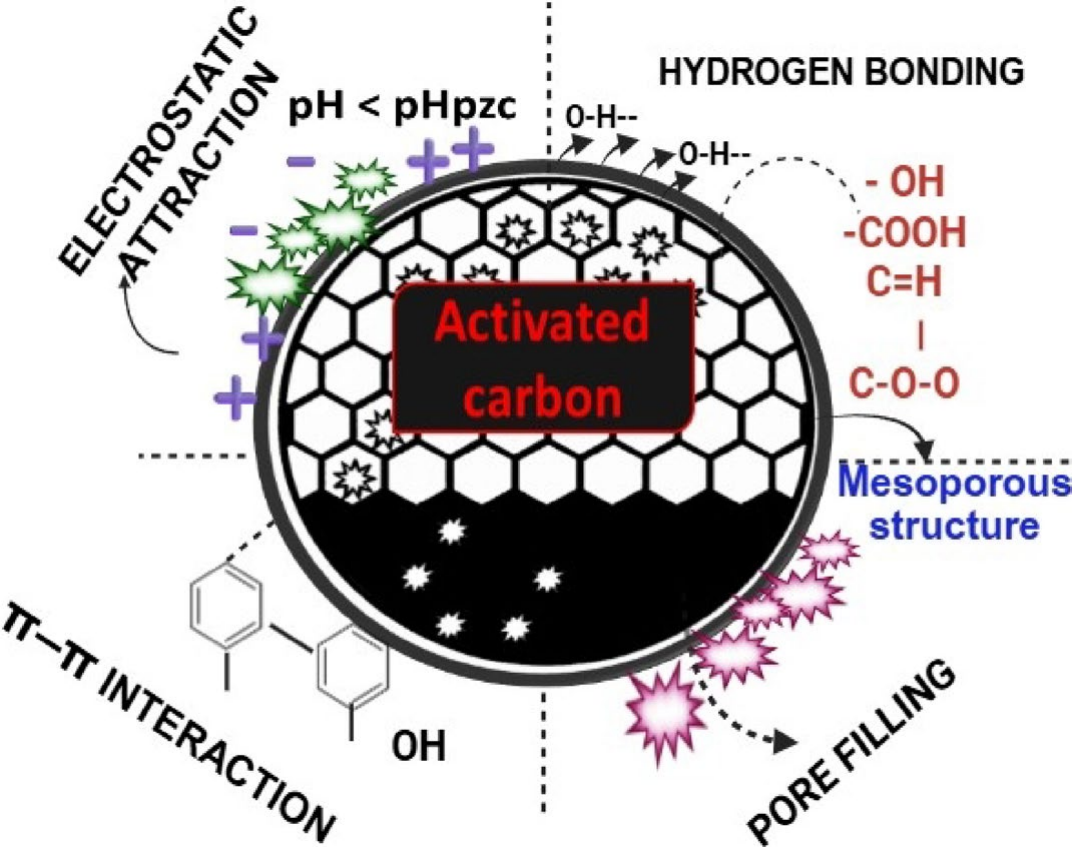
Schematic image showing the interaction mechanisms among BTPAC with phenol.

The schematic image illustrating the interaction mechanisms among the BTPAC and phenol is shown in Fig. 16. Extensive characterization and adsorption results support the idea that phenol adsorption onto the synthesized activated carbon involves a complex interaction between physical and chemical forces. The ultimate analysis reveals a high carbon content, indicating a hydrophobic surface that is ideal for phenol adsorption. With a surface area of 1090 m^2^/g and a dominance of mesopores, rapid diffusion of phenol is facilitated. Broad peaks observed in the XRD analysis confirm an amorphous structure, indicating that the carbon matrix and the aromatic rings of phenol have π–π electron donor–acceptor interactions.

FESEM images show a well-developed porous morphology, and EDS analysis indicates a carbon-rich surface essential for hydrophobic and π–π interactions. The existence of functional groups that contain oxygen is revealed by FTIR and XPS analyses, which also show a noticeable shift or decrease in intensity after adsorption, Verifying the existence of chemical interaction and hydrogen bonding. The pHpzc value and the surface’s acidic nature, as verified by Boehm titration, indicate that the surface becomes positively charged at pH < 5, improving electrostatic attraction to neutral phenol. The Langmuir isotherms are best fitted by the equilibrium values, demonstrating homogeneous surface monolayer adsorption. The PSO kinetic model indicates that chemisorption, potentially involving electron exchange or surface complexation, is the rate-limiting step. Phenol is eliminated by a numerous mechanism that includes pore-filling, π–π assembling, bonding with hydrogen, and pH-dependent electrostatic attraction. Overall, while the adsorption energetics are predominantly driven by physisorption, they are also influenced by chemisorptive features at the active surface sites.

## Conclusion

This study successfully created mesoporous activated carbon from *Bauhinia monandra* tree-derived activated carbon using chemical activation at a relatively low temperature. This material is an inexpensive and locally available agricultural waste. The activated carbon produced has an amorphous, honeycomb-like structure that is well-suited for adsorption applications. It has an abundant surface area of 1090 m^2^/g and a substantial pore volume of 0.539 cm^3^/g. Numerous surface functional groups and well-developed porosity, both of which were verified by characterisation studies, are responsible for its exceptional effectiveness in eliminating phenolic pollutants from aqueous solutions. The point of zero charge results agrees with the Boehm titration analysis. Adsorption studies demonstrated exceptional phenol removal efficiencies and maximal monolayer adsorption capacities of 185.93 mg/g under ideal conditions (dosage of 1.6 g/L, pH 4, 299 K). According to isotherm analysis, the Langmuir isotherm model best describes phenol adsorption and fits the experimental data extremely well. The Langmuir isotherm confirmed monolayer adsorption with a high R^2^ value of 0.99. Adsorption is regulated by chemisorption, where valence forces are intricate through electron sharing or exchange between the activated carbon and the adsorbate, and the rate is determined by the square of the available sites, according to the kinetic study, which validates that the PSO model followed the R^2^ > 0.99. The thermodynamic study suggests that phenol adsorption is more efficient at lower temperatures, from the ΔG° and aligning with its exothermic nature, as confirmed by the calculated enthalpy change (ΔH°) of –17.07 kJ/mol. A negative ΔS° value indicates a decrease in randomness, suggesting that phenol molecules become less flexible as they move from the phenol solution phase to the solid phase of the adsorbent surface. With an activation energy of 25.20 kJ/mol, the adsorption behaviour was shown to be physical. The adsorbent’s heterogeneous surface with a range of active sites with distinct chemical characteristics and pores of different sizes was indicated by the isosteric heat of adsorption. Furthermore, the desorption-regeneration tests maintained their effectiveness across several cycles, underscoring their long-term viability and practical reusability. A synergistic mix of pore diffusion, electrostatic forces, bonding through hydrogen, and π–π interactions could be the adsorption mechanism, according to findings from FTIR, XPS, and other characterisation investigations. The point of future research should concentrate on adsorption efficacy in complicated industrial effluents with mixed organic and inorganic pollutants, performance evaluation in continuous flow systems, and scaling the synthesis process.

## Supplementary Information

Below is the link to the electronic supplementary material.

Supplementary Material 1

## Data Availability

The authors declare that the dataset supporting the findings is available within the article and its accompanying supplementary file. Further enquiries regarding the datasets generated during the current study can be directed to the corresponding author C.R.G.
