# Supplementary material for: Bauhinia monandra derived mesoporous activated carbon for the efficient adsorptive removal of phenol from wastewater
Source: Sci Rep. 2025 Aug 29;15:31790. doi: 10.1038/s41598-025-17444-w (PMC12394648; doi:10.1038/s41598-025-17444-w)
Supplement: Supplementary file 1 — Supplementary Material 1 [file 41598_2025_17444_MOESM1_ESM.docx]

***Bauhinia monandra* derived mesoporous activated carbon for the efficient adsorptive removal of phenol from wastewater**

**Bhojaraja Mohan, Chikmagalur Raju Girish *, Gautham Jeppu, Praveengouda Patil**

Department of Chemical Engineering, Manipal Institute of Technology (MIT), Manipal Academy of Higher Education (MAHE), Manipal, 576104, Karnataka, India

*Corresponding author. E-mail: cr.girish@manipal.edu

# **SUPPLEMENTARY FILE**

**List of Tables**

| **Table S1** | The details of adsorption isotherm models | 3 |
| --- | --- | --- |
| **Table S2** | The details of adsorption kinetic models | 4 |
| **Table S3** | Results of Boehm titration | 4 |
| **Table S4** | The experimental design matrix, along with the experimental and predicted phenol removal percentages | 5 |
| **Table S5** | Adsorption kinetic parameters for phenol adsorption onto BTPAC | 7 |

**List of Figures**

| **Fig. S1** | Absorbance vs wavelength graph for degradation of phenol | 8 |
| --- | --- | --- |
| **Fig. S2** | EDS analysis- Before & After adsorption | 8 |
| **Fig. S3** | Individual and interactive effect of optimization parameters generated from RSM | 9 |
| **Fig. S4** | Effect of the initial phenol concentration on the separation factor (R_L_) | 10 |
| **Fig. S5** | Adsorption kinetics experimental curves with model curves | 11 |

**Table S1: The details of adsorption isotherm models**

| **Isotherm models** | **Non-linear equations** | **References** |
| --- | --- | --- |
| Langmuir | $q_{e}=\frac{q_{m}*K_{L}*C_{e}}{1+K_{L}*C_{e}}$  $R_{L}= 1/ (1+K_{L}* Co)$ | ^43^ |
| Redlich–Peterson | $q_{e}=\frac{A_{RP}*C_{e}}{1+K_{RP}*C_{e}^{B}}$ | ^44^ |
| Temkin | $q_{e}=\frac{RT}{b_{T}}*ln\left( A_{T}*C_{e} \right)$ | ^45^ |
| Freundlich | $q_{e}=K_{F}*C_{e}^{\left( \frac{1}{n} \right)}$ | ^46^ |
| Dubinin–Radushkevich | $q_{e}=q_{max}e^{{-\beta r.є}^{2}}$  $є=RTln(1+\frac{1}{C_{e}})$ | ^47^ |

Wherein, $q_{m}$​ represents the maximum adsorption capacity of phenol per unit mass of BTPAC, while $C_{e}$*​* denotes the equilibrium aqueous concentration of phenol. $K_{L}$*​,* $K_{F}$ *​,* $A_{RP}$ *,* $K_{RP}$ *​,* $B$*,* $A_{T}$​and $B_{T}$​ are constants associated with the Langmuir, Freundlich, Redlich-Peterson, and Temkin isotherms models, respectively. Additionally, $\beta r$ is the Dubinin-Radushkevich constant, and $R$is the universal gas constant (8.31 J/mol K).

**Table S2: The details of adsorption kinetics models**

| **Kinetic models** | **Non-linear equations** | **References** |
| --- | --- | --- |
| Pseudo first-order | $q_{t}=q_{e}*\left( 1-e^{-k_{1}t} \right)$ | ^48^ |
| Pseudo-second-order | $q_{t}=\left( \frac{k_{2}*q_{e}^{2}*t}{1+k_{2}*q_{e}*t} \right)$ | ^49^ |
| Elovich | $q_{t}=\left( \frac{1}{b} \right)ln(1+abt)$ | ^50^ |
| Intraparticle diffusion | ${q_{t}=K}_{diff}t^{0.5}+C$ | ^51^ |

wherein, $q_{t}$ and $q_{e}$ represents adsorption capacity at time $t$ and equilibrium, correspondingly. $k_{1}$ *,* $k_{2}$*,* and $K_{diff}$ represent the respective Pseudo first-order, Pseudo-second-order and Intraparticle diffusion (IPD) rate constant values. *C* is the IPD intercept. *a* is the initial adsorption rate (mg/g min), and *b* is the desorption constant (g/mg) in the Elovich model.

**Table S3: Results of Boehm titration**

| **Functional groups** | **Carboxylic groups** | **Lactonic groups** | **Phenolic** | **Total acidic groups** | **Total basic groups** |
| --- | --- | --- | --- | --- | --- |
| **Amount**  **(*meq/g*)** | 0.389 | 0.12 | 0.81 | 1.317 | 0.038 |

**Table S4: The experimental design matrix, along with the experimental and predicted phenol removal percentages**

| Run | Design points | Optimization parameters | | | | Phenol removal  Percentage | |
| --- | --- | --- | --- | --- | --- | --- | --- |
|  |  | Dosage  (A) | pH  (B) | Temperature  (C) | Initial  concentration  (D) | Experimenl | Predicted |
| 1 | Factorial | 1.8 | 9 | 45 | 40 | 49.81 | 49.86 |
| 2 |  | 1.8 | 4 | 25 | 40 | 65.63 | 66.16 |
| 3 |  | 0.6 | 4 | 25 | 40 | 32.08 | 32.38 |
| 4 |  | 1.8 | 4 | 45 | 40 | 53.82 | 54.11 |
| 5 |  | 0.6 | 4 | 45 | 20 | 33.01 | 33.63 |
| 6 |  | 0.6 | 9 | 45 | 20 | 31.43 | 30.83 |
| 7 |  | 1.8 | 4 | 25 | 20 | 83.9 | 84.59 |
| 8 |  | 0.6 | 9 | 25 | 40 | 29.59 | 29.48 |
| 9 |  | 1.8 | 9 | 25 | 20 | 81.4 | 82.2 |
| 10 |  | 0.6 | 4 | 25 | 20 | 42.65 | 42.53 |
| 11 |  | 0.6 | 9 | 25 | 20 | 40.9 | 40.62 |
| 12 |  | 0.6 | 9 | 45 | 40 | 19.81 | 19.13 |
| 13 |  | 1.8 | 9 | 25 | 40 | 63.4 | 62.79 |
| 14 |  | 0.6 | 4 | 45 | 40 | 23.77 | 22.9 |
| 15 |  | 1.8 | 4 | 45 | 20 | 73.07 | 73.11 |
| 16 |  | 1.8 | 9 | 45 | 20 | 70.13 | 69.84 |
| 17 | Axial | 1.2 | 6.5 | 20.857 | 30 | 66.15 | 65.28 |
| 18 |  | 0.3514 | 6.5 | 35 | 30 | 17.13 | 18.33 |
| 19 |  | 1.2 | 6.5 | 49.142 | 30 | 48.85 | 49.84 |
| 20 |  | 1.2 | 2.96447 | 35 | 30 | 59.51 | 58.43 |
| 21 |  | 1.2 | 6.5 | 35 | 44.14 | 37.1 | 37.86 |
| 22 |  | 1.2 | 6.5 | 35 | 15.85 | 59.81 | 59.17 |
| 23 |  | 1.2 | 10.0355 | 35 | 30 | 52.87 | 54.07 |
| 24 |  | 2.0485 | 6.5 | 35 | 30 | 70.89 | 69.8 |
| 25 | Center | 1.2 | 6.5 | 35 | 30 | 53.62 | 53.56 |
| 26 |  | 1.2 | 6.5 | 35 | 30 | 53.06 | 53.56 |
| 27 |  | 1.2 | 6.5 | 35 | 30 | 52.3 | 53.56 |
| 28 |  | 1.2 | 6.5 | 35 | 30 | 55.09 | 53.56 |
| 29 |  | 1.2 | 6.5 | 35 | 30 | 54.36 | 53.56 |
| 30 |  | 1.2 | 6.5 | 35 | 30 | 53.17 | 53.56 |

Table S5: Adsorption kinetic parameters for phenol adsorption onto BTPAC.

| $\boldsymbol{C}\boldsymbol{o}$  **(mg/L)** | $\boldsymbol{q}_{\boldsymbol{exp}}$  **(mg/g)** | **Pseudo-first-order kinetic model** | | | | **Pseudo-second-order kinetic model** | | | | |
| --- | --- | --- | --- | --- | --- | --- | --- | --- | --- | --- |
|  |  | $k_{1}$  (min^-1^) | $q_{e.model}$  (mg/g) | $R^{2}$ | SSE | $k_{2}$  (min^-1^) | $q_{e.model}$(mg/g) | $R^{2}$ | $\chi^{2}$ | SSE |
| 30 | 13.88 | 0.42 | 13.39 | 0.94 | 15.69 | 0.04 | 13.92 | 0.98 | 0.16 | 2.64 |
| 60 | 25.60 | 0.46 | 24.73 | 0.95 | 39.94 | 0.02 | 25.73 | 0.99 | 0.26 | 4.21 |
| 90 | 37.42 | 0.31 | 36.34 | 0.95 | 109.0 | 0.01 | 37.98 | 0.98 | 1.83 | 29.32 |
| 120 | 44.68 | 0.28 | 44.06 | 0.97 | 75.1 | 0.008 | 46.21 | 0.98 | 2.44 | 39.11 |
| 150 | 53.93 | 0.21 | 52.10 | 0.93 | 340.3 | 0.005 | 54.88 | 0.96 | 9.73 | 155.7 |

| $\boldsymbol{C}\boldsymbol{o}$  **(mg/L)** | $\boldsymbol{q}_{\boldsymbol{exp}}$  **(mg/g)** | **Intraparticle**  **diffusion kinetic model** | | | **Elovich Kinetic Model** | | |
| --- | --- | --- | --- | --- | --- | --- | --- |
|  |  | $k_{diff}$  ((mg/g). min^0.5^) | *C* (mg/g) | $R^{2}$ | *a*  (mg. (g min)^-1^) | *b*  (g.mg^-1^) | $R^{2}$ |
| 30 | 13.88 | 0.65 | 6.98 | 0.60 | 159.24 | 0.65 | 0.94 |
| 60 | 25.60 | 1.16 | 13.32 | 0.57 | 159.24 | 0.65 | 0.95 |
| 90 | 37.42 | 1.9 | 16.75 | 0.65 | 397.78 | 0.36 | 0.93 |
| 120 | 44.68 | 2.42 | 19.18 | 0.60 | 66.86 | 0.01 | 0.94 |
| 150 | 53.93 | 3.10 | 20.27 | 0.72 | 123.97 | 0.16 | 0.92 |

**List of Figures**

**
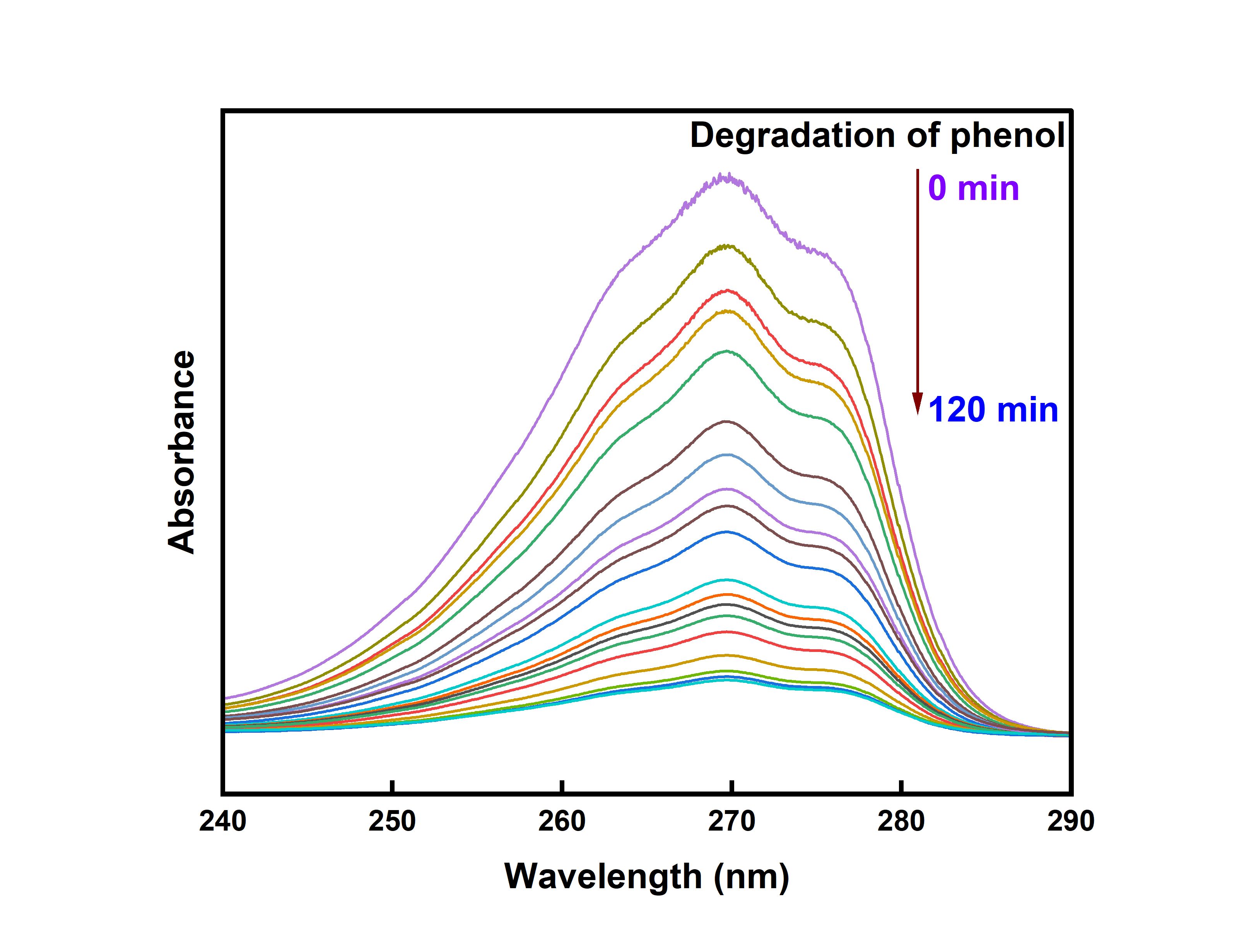
**

**Fig. S1: Absorbance vs wavelength graph for degradation of phenol**

| 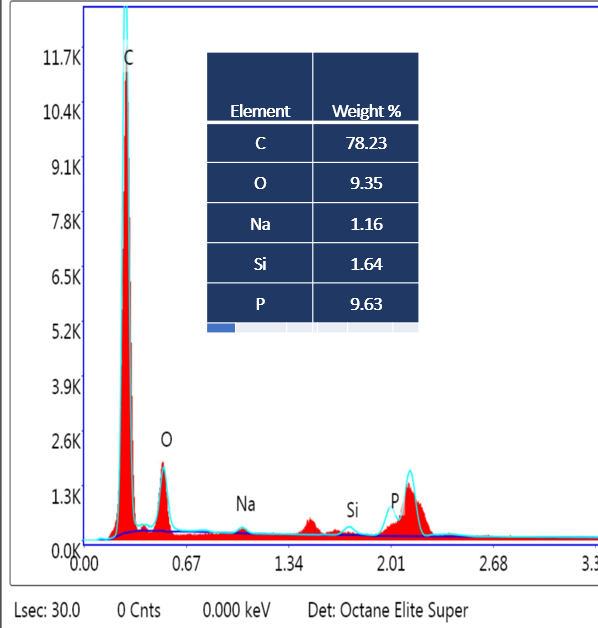 | 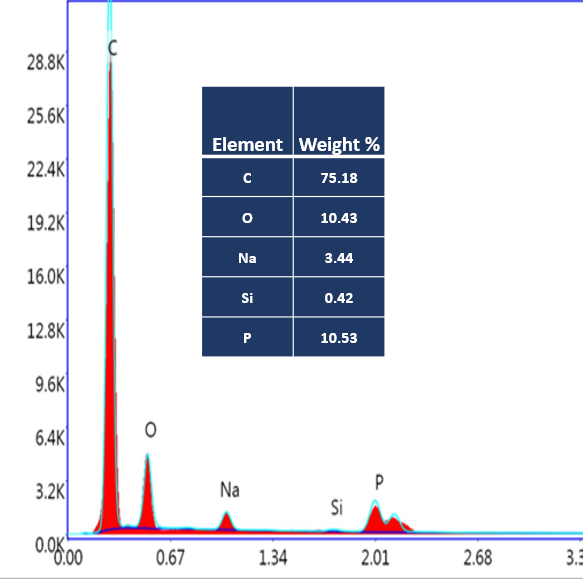 |
| --- | --- |

**Fig. S2: EDS analysis- before and after adsorption of BTPAC**

| 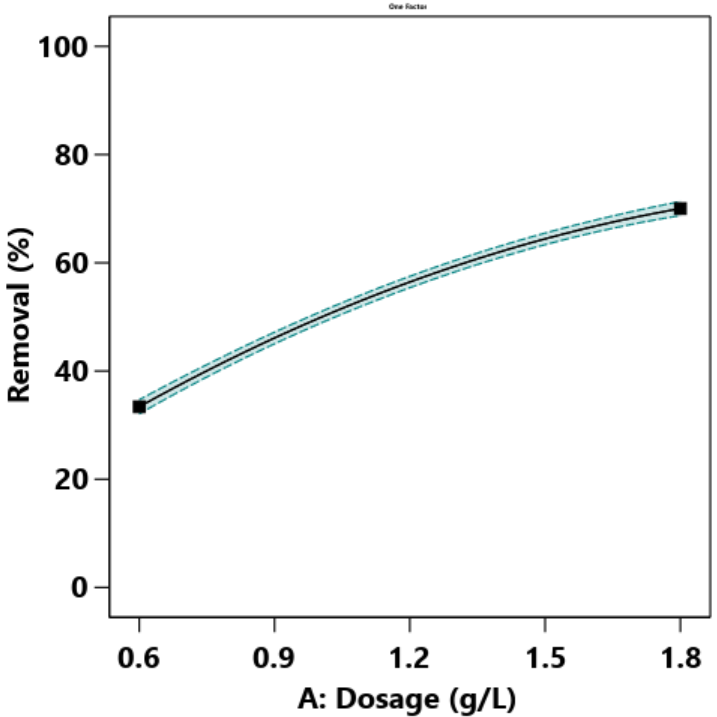 | 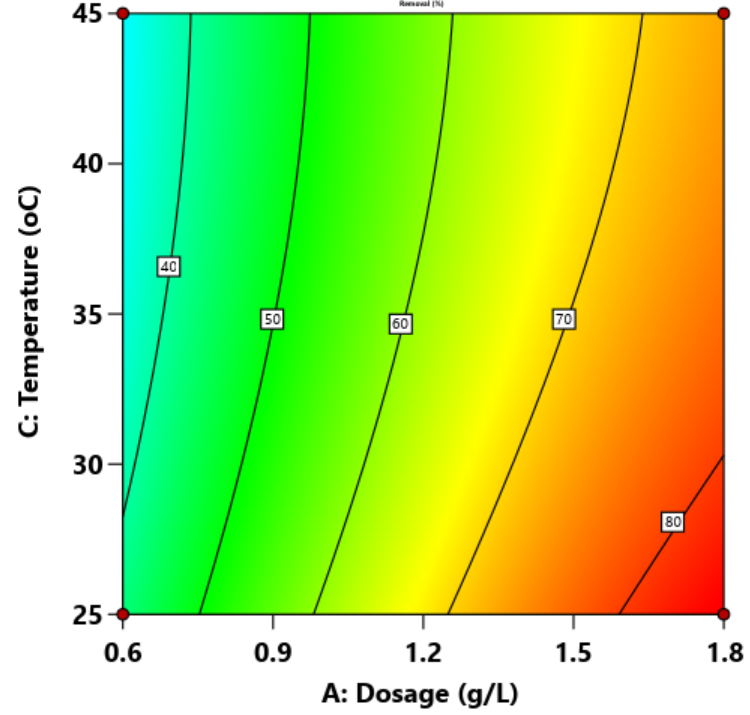 | |
| --- | --- | --- |
| **(a)** | **(b)** | |
| 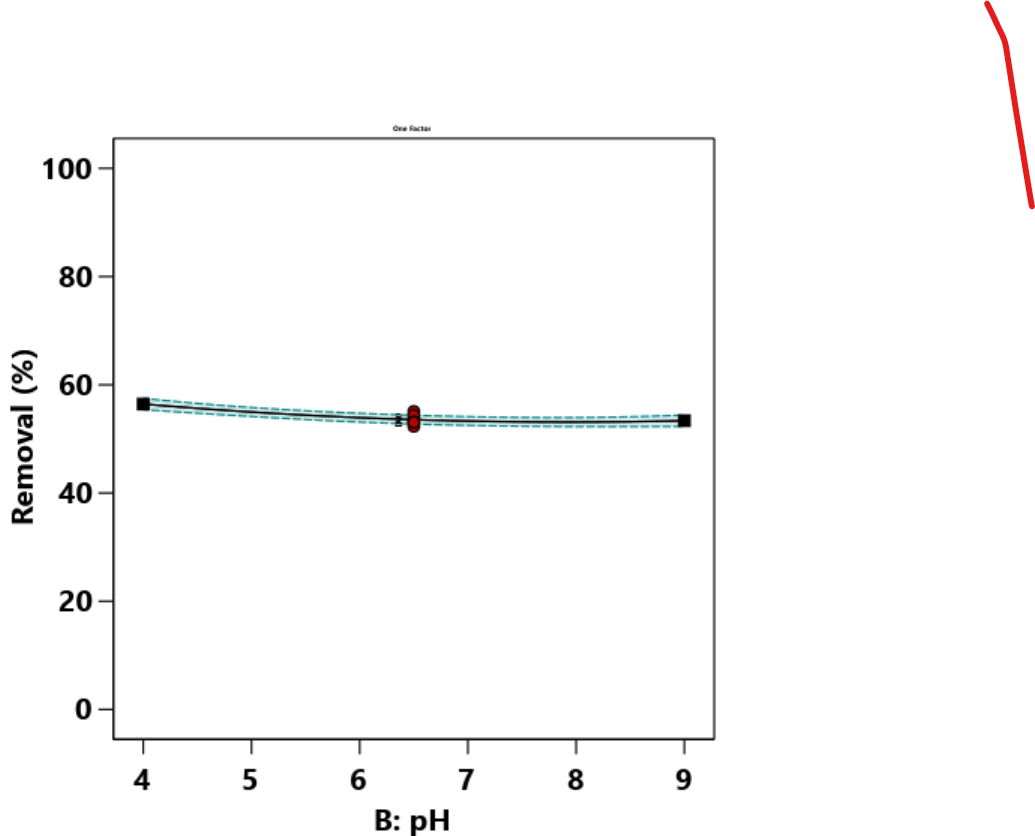 | 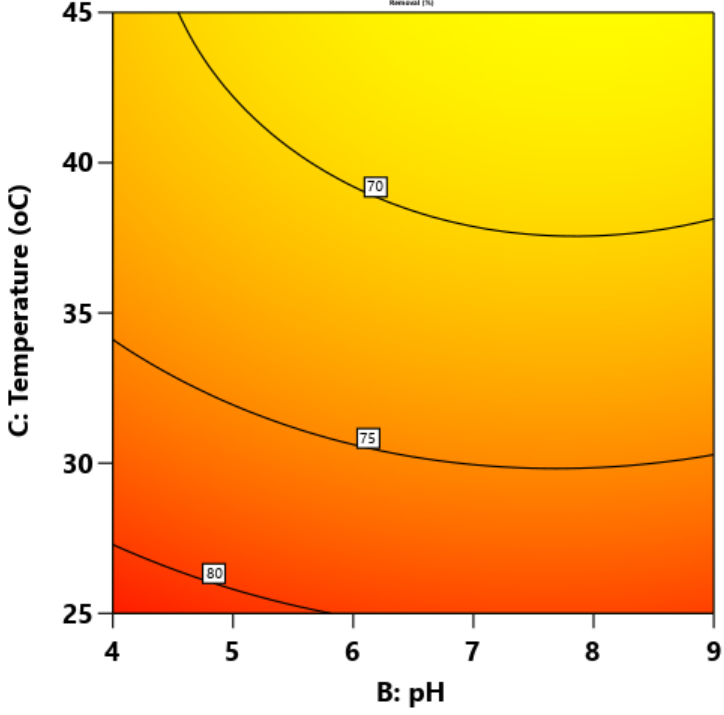 | |
| **(c)** | **(d)** | |
| 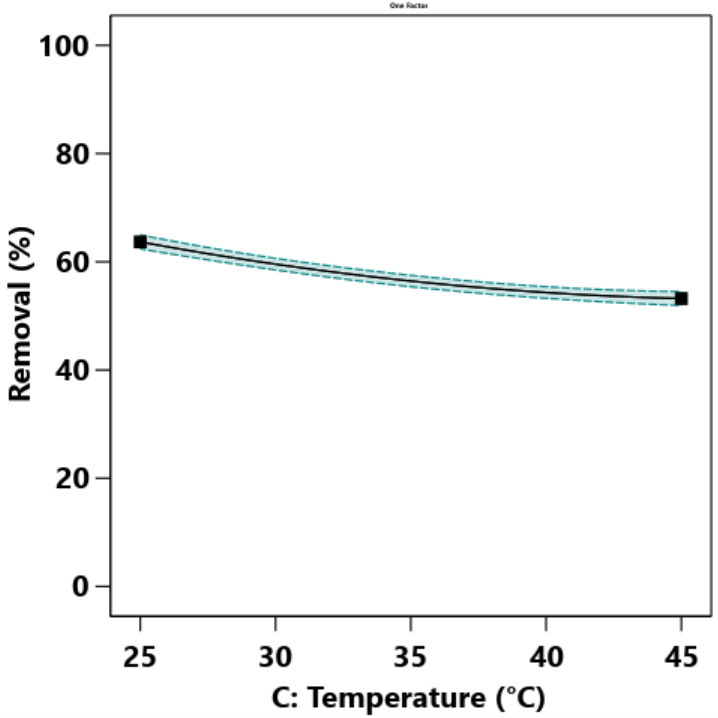 | | 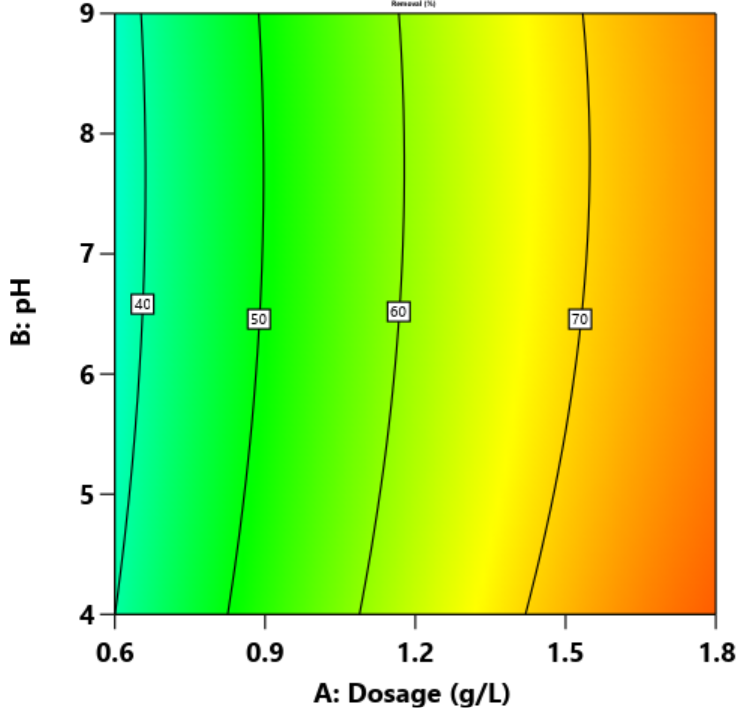 |
| **(e)** | | **(f)** |
| 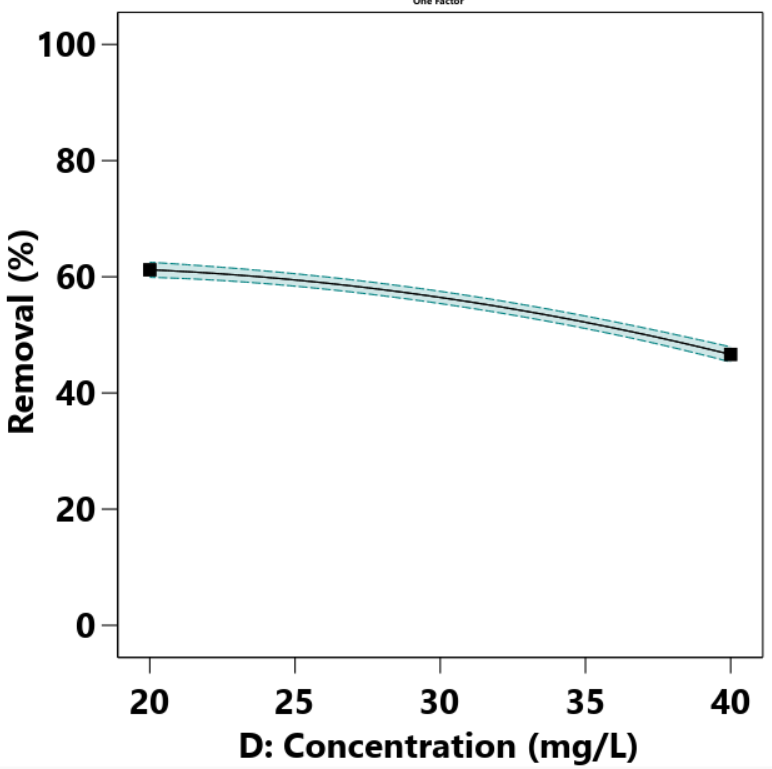 | | 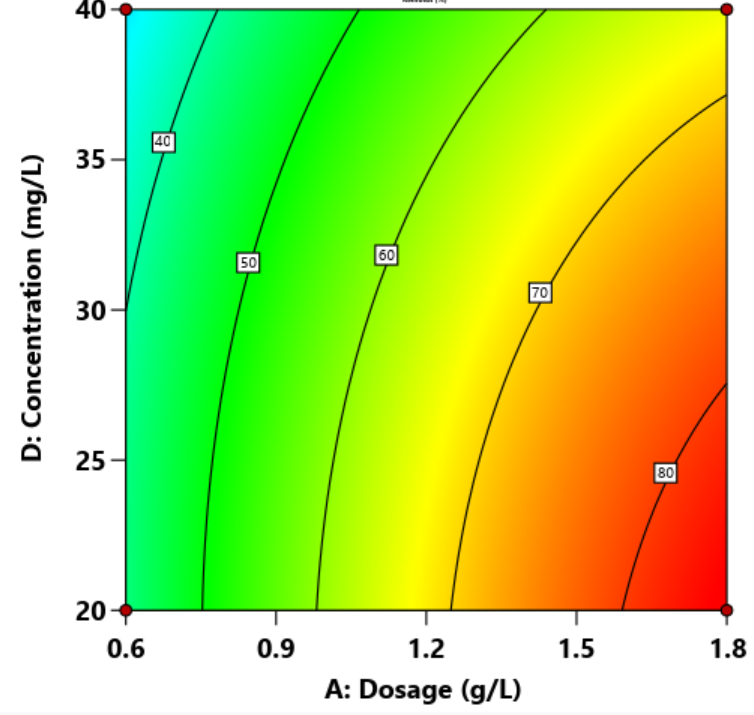 |
| **(g)** | | **(h)** |

Fig. S3: One factor graph (a) Dosage, (c) pH, (e) Temperature, (g) Initial concentration and Contour plots of phenol removal showing interactive effects of (b) Dosage and pH, (d) pH and Temperature, (f) Dosage and temperature, (g) Temperature and concentration


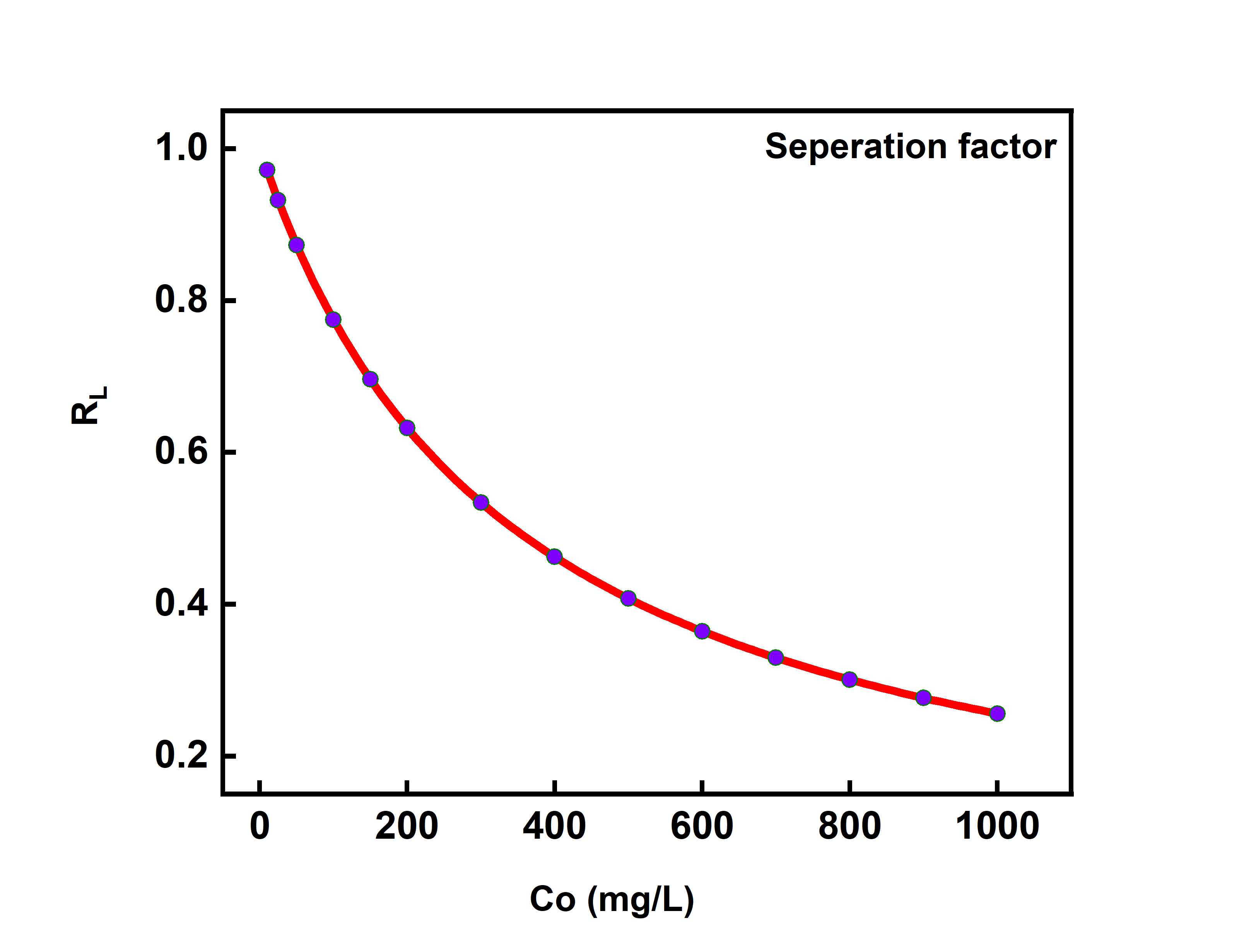


**Fig. S4: Effect of the initial phenol concentration on the** **separation factor (R_L_)**

| **** |  |
| --- | --- |
| **(a)** | **(b)** |
|  |  |
| **(c)** | **(d)** |

Fig. S5: Adsorption kinetic experimental curves with model curves:

(a) Pseudo-First-Order Kinetic Model (b) Pseudo-Second-Order Kinetic Model

(c) Intraparticle Diffusion Model. (d) Elovich Kinetic Model

***
